# Combining tissue biomarkers with mpMRI to diagnose clinically significant prostate cancer. Analysis of 21 biomarkers in the PICTURE study

**DOI:** 10.1038/s41391-024-00920-1

**Published:** 2024-11-22

**Authors:** Urszula Stopka-Farooqui, Vasilis Stavrinides, Benjamin S. Simpson, Hania Qureshi, Lina M. Carmona Echevierra, Hayley Pye, Zeba Ahmed, Mohammed F. Alawami, Jonathan D. Kay, Jonathan Olivier, Susan Heavey, Dominic Patel, Alex Freeman, Aiman Haider, Caroline M. Moore, Hashim U. Ahmed, Hayley C. Whitaker

**Affiliations:** 1 Division of Surgery and Interventional Science, University College London, London, UK; 2 Department of Urology, UCLH NHS Foundation Trust, London, UK; 3 Department of Urology, Hospital Huriez, University Lille Nord de France, Lille, France; 4 Department of Pathology, UCLH NHS Foundation Trust, London, UK; 5 Division of Surgery, Department of Surgery and Cancer, Faculty of Medicine, Imperial College London, London, UK; 6 Imperial Urology, Charing Cross Hospital, Imperial College Healthcare NHS Trust, London, UK

**Keywords:** Prostate cancer, Diagnostic markers, Prognostic markers, Prostate cancer

## Abstract

**Background:**

Serum PSA and digital rectal examination remain the key diagnostic tools for detecting prostate cancer. However, due to the limited specificity of serum PSA, the applicability of this marker continues to be controversial. Recent use of image-guided biopsy along with pathological assessment and the use of biomarkers has dramatically improved the diagnosis of clinically significant cancer. Despite the two modalities working together for diagnosis biomarker research often fails to correlate findings with imaging.

**Methods and results:**

We looked at 21 prostate cancer biomarkers correlating our results with mpMRI data to investigate the hypothesis that biomarkers along with mpMRI data make a powerful tool to detect clinically significant prostate cancer. Biomarkers were selected based on the existing literature. Using a tissue microarray comprised of samples from the PICTURE study, with biopsies at 5 mm intervals and mpMRI data we analysed which biomarkers could differentiate benign and malignant tissue. Biomarker data were also correlated with pathological grading, mpMRI, serum PSA, age and family history. AGR2, CD10 and EGR protein expression was significantly different in both matched malignant and benign tissues. AMACR, ANPEP, GDF15, MSMB, PSMA, PTEN, TBL1XR1, TP63, VPS13A and VPS28 showed significantly different expression between Gleason grades in malignant tissue. The majority of the biomarkers tested did not correlate with mpMRI data. However, CD10, KHDRBS3, PCLAF, PSMA, SIK2 and GDF15 were differentially expressed with prostate cancer progression. AMACR and PTEN were identified in both pathological and image data evaluation.

**Conclusions:**

There is a high demand to develop biomarkers that would help the diagnosis and prognosis of prostate cancer. Tissue biomarkers are of particular interest since immunohistochemistry remains a cheap, reliable method that is widely available in pathology departments. These results demonstrate that testing biomarkers in a cohort consistent with the current diagnostic pathway is crucial to identifying biomarker with potential clinical utility.

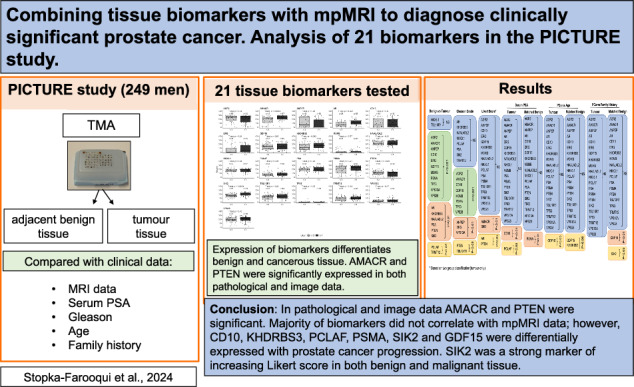

## Introduction

An early and accurate diagnosis of prostate cancer (PCa), and its timely treatment, are the key components in managing the disease and outcome. Traditionally, the measurement of serum prostate-specific antigen (PSA), alongside digital rectal exam, is used as gold standard in PCa detection [1, 2]. More recently the use of multiparametric MRI imaging (mpMRI) with image-guided biopsy, Gleason grading and clinical stage has contributed enormously to the improved diagnosis and risk stratification of PCa [3, 4]. As a minimally invasive technique, mpMRI has also eliminated unnecessary biopsies and supported individual treatment plans that require either more aggressive therapies or active surveillance. There are limited data available for biomarkers in conjunction with imaging, even though it plays an increasingly important role in the diagnostic pathway.

PCa is a multifocal, heterogenous disease making accurate diagnosis and prognosis challenging. This results in a demand for better diagnostic tools to accurately identify those cancers that will limit survival and to stratify those patients that can be safely managed with active surveillance [5, 6]. Fluidic and tissue-based diagnostic biomarkers are increasingly abundant. Despite the plethora of tests available, many are not cost-effective and have not been routinely adopted [5, 7–10]. Meanwhile, immunohistochemistry (IHC) remains routinely performed in the majority of pathology laboratories and offers a cost-effective method for improving diagnosis using clinical samples that are routinely available.

The work presented here investigates 21 biomarkers selected through the literature searches and unpublished data obtained in our laboratory. We focused on markers previously associated with PCa; however, many of them are also associated with other cancers (Table 1).

**Table 1:** Summary of tissue biomarkers used in the study.

Protein symbol	Protein name	Alias	Protein accession code	Expression in cancer	Protein expression in prostate cancer	Image (MRI) data available	Reference
AGR2	Anterior Gradient 2	GOB-4	O95994	breast, prostate, oesophageal, pancreatic, colorectal, lung, hepatocellular carcinoma	↑	NO	[32–35]
AMACR	Alpha-Methylacyl-CoA Racemase	P504S	Q9UHK6	breast, prostate, colorectal, ovarian, bladder, lung, lymphoma, renal cell carcinomas, melanoma	↑	YES	[11, 36–39]
ANPEP	Alanyl Aminopeptidase	CD13	P15144	breast, prostate, gallbladder, leukaemia, ovarian, thyroid, pancreatic	↓	NO	[40–43]
AR	Androgen Receptor	NR3C4	P10275	breast, prostate, oesophageal, gastric, liver, pancreatic	↑	NO	[44–46]
CD10	Cluster of differentiation 10/ membrane metalloendopeptidase	MME	P08473	breast, prostate, leukaemia, renal, bladder, lung, colorectal,	↑↓	NO	[23, 47, 48]
ERG	Transcriptional Regulator ERG protein	TMPRSS2-ERG fusion	P11308	colorectal, prostate, leukaemia	↑	YES	[38, 49–52]
GDF15	Growth Differentiation Factor 15	MIC-1	Q99988	breast, prostate, colorectal	↑	YES	[53–55]
KHDRBS3	KH RNA Binding Domain Containing, Signal Transduction 3	TSAR	O75525	breast, prostate, colorectal, ovarian	↑	NO	[56–58]
MSMB	Microseminoprotein Beta	PSP-94	P08118	prostate	↓	YES	[11, 59]
PCLAF	PCNA Clamp Associated Factor	KIAA0101	Q15004	breast, prostate, adrenal, thyroid, pancreatic, gastric, lung, ovarian,	↑	NO	[60–63]
NAALADL2	N-Acetylated Alpha-Linked	NAALADase L2	Q58DX5	colon, prostate	↑	NO	[64–66]
NKX3.1	Homeobox Protein NK-3 Homologue A	BAPX2	Q99801	breast, prostate, nasopharyngeal	↓	NO	[67–69]
PSA	Prostate-specific antigen	KLK3	P07288	breast, prostate, ovarian,	↑	YES	[11, 38, 70, 71]
PSMA	Prostate-specific Membrane Antigen	FOLH	Q04609	endothelium of tumour-associated neovasculature of breast, prostate, lung and urothelial cancer	↑	YES	[11, 72, 73]
PTEN	Phosphatase And Tensin Homologue	MMAC 1	P60484	breast, prostate, colon, lung, glioblastoma, ovarian, kidney	↓	YES	[49, 51, 67, 74–77]
SIK2	Salt Inducible Kinase 2	SNF1LK2	Q9H0K1	breast, prostate, ovarian, lymphoma	↑	NO	[29, 78]
TBL1XR1	Transduction-Beta-Like 1 x-linked Receptor 1	TBLR1	Q9BZK7	breast, prostate, ovarian, gastric, colorectal, liver, lymphoma, lung	↑	NO	[64, 79, 80]
TP63	Tumour protein p63	P63	Q9H3D4	breast, prostate, vulvar, skin, bladder	↓	YES	[11, 81, 82]
TRMT12	TRNA Methyltransferase 12 Homologue	TYW2	Q53H54	breast, prostate, neck	↑	NO	[83, 84]
VPS13A	Vacuolar Protein Sorting 13 Homologue A	Chorein	Q96RL7	gastric, colorectal, ovarian, testis, prostate	↑	NO	[85–87]
VPS28	Vacuolar Protein Sorting Associated Protein 28 Homologue	ESCRT-I complex subunit VPS28	Q9UK41	breast, lung, liver, prostate	↑	NO	[88–91]

Protein symbol, alias and accession code are taken from UniProt. The arrows represent the upregulation (↑) and downregulation (↓) reported in PCa. Image (MRI) data stating ‘YES’ represents biomarkers where image data are already published in other studies, while ‘NO’ refers to image data not available in previous studies.

Biomarkers were analysed in a cohort of 249 patients from the PICTURE study, utilising a tissue microarray (TMA) comprising malignant biopsy cores paired with matched adjacent benign tissue for each patient, resulting in a total of 449 core sections per patient. These results were conducted in conjunction with mpMRI and diagnostic clinical data. Notably, many of the biomarkers investigated in this study provide the first dataset to be examined alongside imaging data or provide additional validation in a larger dataset. Additionally, our analysis encompassed the evaluation of pathological Gleason scores and biopsy grade groups (GG) to determine the correlation between the clinically significant and clinically insignificant disease with image data. This comprehensive approach allows for a detailed understanding of PCa pathology, combining traditional histopathological grading with emerging biomarker insights and imaging modalities.

## Material and methods

### Tissue microarray

The TMA is comprised of biopsy cores from 249 patients enroled in the PICTURE trial that investigated the accuracy of mpMRI to diagnose clinically significant prostate cancer (csPCa) [11, 12]. csPCa was defined based on TPM (template prostate mapping) biopsy, Gleason grade and maximum cancer core length (MCCL) measures. The primary definition of significance was based on the presence of dominant Gleason pattern 4 or greater (Gleason ≥4 + 3) and/or a MCCL of ≥6 mm. This was the primary objective of the study. Further to this, a definition two for the secondary outcome analysis (Gleason ≥3 + 4 and/or MCCL ≥ 4 mm) as well as the presence of any Gleason score 7 or more was used. Clinically insignificant disease was defined as Gleason pattern 3 and MCCL ≤ 3 mm. Therefore, tissue with the highest pathological Gleason score and/or the longest MCCL was used to construct the TMA as described [11]. There were three categories of samples: true benign (patients whose tissue was assessed as benign, no cancer), malignant tissue (patients diagnosed with PCa) and adjacent, benign tissue (sometimes refer as matched benign tissue; a neighbouring benign tissue that comes from the same biopsy core as malignant sample). A summary of patient clinical data can be found in Supplementary Table 1. Ethics committee approval for PICTURE was granted by London City Road and Hampstead National Research Ethics Committee (11/LO/1657) and all procedures were in accordance with the Helsinki Declaration. Informed consent was obtained from all individual participants included in the study.

### Biomarker selection

Biomarkers were identified through literature searches and prioritised based upon published data that was indicative of a role in diagnosis in PCa as well as the availability of high-quality reagents for IHC. Other markers were selected as they form part of ongoing experiments and unpublished data obtained within the laboratory. A list of the biomarkers tested can be found in Table 1.

### Immunohistochemistry

IHC was performed using a Leica BOND-MAX Autostainer (Leica Biosystems, Milton Keynes, UK). Dewaxing and rehydration took planning manually in advance. Antibodies were optimised using imperfect TMA slides and stained with antibodies diluted in either primary antibody diluent (Leica Biosystems, Milton Keynes, UK) or a high stringency diluent for enhanced target specificity (10× Tris buffered-saline (TBS)) (Trizma Base, Sigma-Aldrich, Gillingham, UK), 10% donkey serum (Sigma-Aldrich, Gillingham, UK) and 1% Tween (Fisher Scientific, Loughborough, UK). Heat- induced epitope retrieval was performed with either Epitope Retrieval solution 1 (ER1, pH 6.0, Leica Biosystems, Milton Keynes, UK) or Epitope Retrieval solution 2 (ER2, pH 9.0, Leica Biosystems, Milton Keynes UK).

Full-face TMA slides were stained for 21 biomarkers and an overview of the optimised conditions can be found in Table 2. Diaminobenzidine (DAB) was used as the chromogen (brown) and haematoxylin used to counterstain the nuclei (blue). Slides underwent manual dehydration before they were cover-slipped with DPX (Merck Millipore, Gillingham, UK).

**Table 2:** Parameters of antibodies used for staining TMAs with BOND-MAX autostainer.

Antibody target	Antibody type	Dilution	Antigen retrieval method	Company	Cat. number
AGR2	Rabbit polyclonal	1:200	ER2 for 20’	Cell Signalling, UK	8992
AMACR	Rabbit polyclonal	1:100	ER1 for 20’	Dako A/S, Denmark	M3616
ANPEP	Mouse monoclonal	1:100	ER1 for 20’	Leica Biosystems, UK	NCL-CD13-304
AR	Mouse monoclonal	1:400	ER1 for 20’	Dako A/S, Denmark	M3562
CD10	Mouse monoclonal	1:50	ER1 for 30’	Leica Biosystems, UK	NCL-CD10-270
ERG	Rabbit monoclonal	1:100	ER1 for 20’	Abcam, UK	ab92513
GDF15	Rabbit polyclonal	1:25	ER1 for 20’	Atlas Antibodies, Sweden	HPA011191
KHDRBS3	Rabbit polyclonal	1:400	ER2 for 20’	Atlas Antibodies, Sweden	HPA000275
PCLAF	Mouse monoclonal	1:100 (high stringency diluent)	ER2 for 20’	Abcam, UK	ab56773
MSMB	Rabbit polyclonal	1:2500	Enzymatic pre-digestion ER1 for 15’	Abcam, UK	ab196754
NAALADL2	Rabbit polyclonal	1:25 (high stringency diluent)	ER2 for 20’	Atlas Antibodies, Sweden	HPA012413
NKX3.1	Rabbit polyclonal	1:300	ER1 for 20’	Athena ES, USA	317
TP63	Mouse monoclonal	1:50	ER2 for 20’	Leica Biosystems, UK	NCL-L-P63
PSA	Rabbit polyclonal	1:9000	no retrieval	Dako A/S, Denmark	A0562
PSMA	Mouse monoclonal	1:50	ER1 for 20’	Leica Biosystems, UK	NCL-L-PSMA
PTEN	Mouse monoclonal	1:100	ER1 for 20’	Dako A/S, Denmark	M3627
SIK2	Goat polyclonal	1:300 (high stringency diluent)	ER1 20’	R&D Systems, USA	AF5737
TBL1XR1	Rabbit monoclonal	1:400	ER1 for 20’	Abcam, UK	Ab190796
TRMT12	Rabbit polyclonal	1:25 (high stringency diluent)	ER1 for 20’	Atlas Antibodies, Sweden	HPA023939
VPS13A	Rabbit polyclonal	1:25	ER1 for 20’	Atlas Antibodies, Sweden	HPA021662
VPS28	Rabbit polyclonal	1:400	ER1 for 20’	Atlas Antibodies, Sweden	HPA024745

Antibodies diluted with high stringency diluent are listed in the table, otherwise Leica primary antibody diluent was used. Epitope Retrieval Solution 1 (ER1) and Epitope Retrieval Solution 2 (ER2) were used with different time points, specified in minutes (’).

### Immunohistochemistry scoring

Slides were digitised using a Hamamatsu RS 2.0 scanner and viewed using the NDP.view software. All scoring was performed blindly in pairs by HW and one other researcher (USF, VS, ZA, BS, MA, HQ, JK, JO, and LMCE). The proportion of positively stained epithelial cells (0–100%) was assessed in the cancer and benign tissue and the intensity of the staining assessed (0 = none, 1 = weak, 2 = medium and 3 = strong). A final score ranging from 0-300 was carried out by using the histochemical scoring (h-score) assessment [13, 14] with the following formula: (percentage of score 3 × 3) + (percentage of score 2 × 2) + (percentage of score 1 × 1).

A total of 449 core segments were stained using IHC against 21 biomarkers, resulting in ~9427 stained core sections. A summary of these findings is presented in Supplementary Tables 2 and 3. Out of these 532 (5.6%) cores were stromal, and 1358 (14.4%) were either lost during the staining process or deemed unassessable due to poor tissue quality.

Where both tumour and benign tissue were present in a core only tumour tissue was scored. Cores identified as stromal, unassessable (poor quality or inadequate tissue present) and/or missing were excluded from further analysis. Following the exclusion, 7537 (79.9%) cores were included in the analysis with 6565 (87.1%) cores showing agreement in appearance on IHC and H&E originally intended (‘as assigned’).

A small proportion of cores needed to be re-assigned as benign or tumour compared to the initial pathological evaluation but the tissue comes from the same core (Supplementary Tables 2 and 3). The pathology of a proportion of cores, or cores where the pathology was ambiguous, was double-checked by uropathologist AF and AH. All Gleason scores were provided by AF and AH.

### Data analysis and statistics

Statistical analysis was performed using the R programme (http://www.R-project.org/, version 4.0.2). The h-score of matched data identified as tumour and adjacent benign (tissue present in the same core with cancer tissue) was analysed alongside additional clinical data e.g. pathological Gleason grade, Likert score, serum PSA, age, ethnicity and family history. For the matched data analysis, the paired Wilcoxon signed rank test was used due to the non-normal distribution of the data. For comparison of multiple groups, the Kruskal-Wallis analysis of variation with the Wilcoxon signed rank test and Dunn test with Bonferroni p-adjustment were used. The statistical significance level of *p* < 0.05 was considered significant.

## Results

### Expression of biomarkers differentiates benign and cancerous tissue

**Fig. 1:**
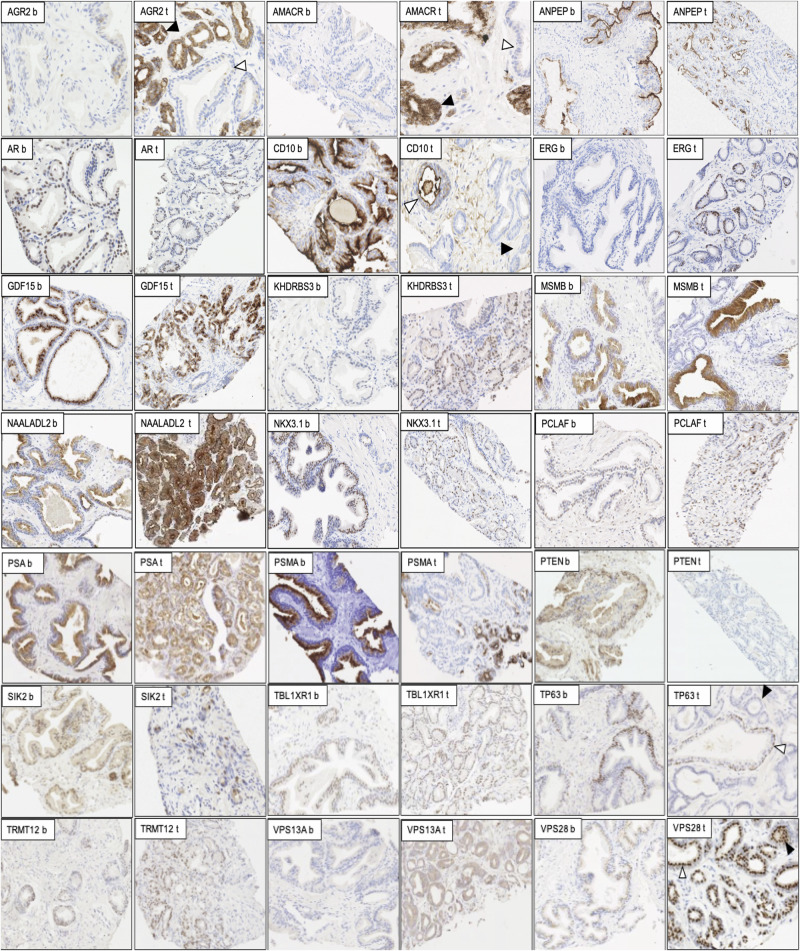
Representative histology of paired tumour and benign-appearing prostatic tissue samples for 21 biomarkers. All prostatic tissue slices were processed, stained using BOND-MAX Autostainer, and reviewed to assess the staining intensity of each biomarker in benign (b) and tumour (t) tissue. The staining of AGR2, AMACR, ANPEP, AR, CD10, ERG, GDF15, KHDRBS3, MSMB, PCLAF, NAALADL2, NKX3.1, PSA, PSMA, PTEN, SIK2, TBL1XR1, TP63, TRMT12, VPS13A and VPS28 are shown in brown and nuclei shown in blue. Full details of all staining conditions can be found in Table 1. Each marker was scored using the h-score method. The white and black arrowheads in some examples refer to benign and tumour tissues in a malignant core.

**Fig. 2:**
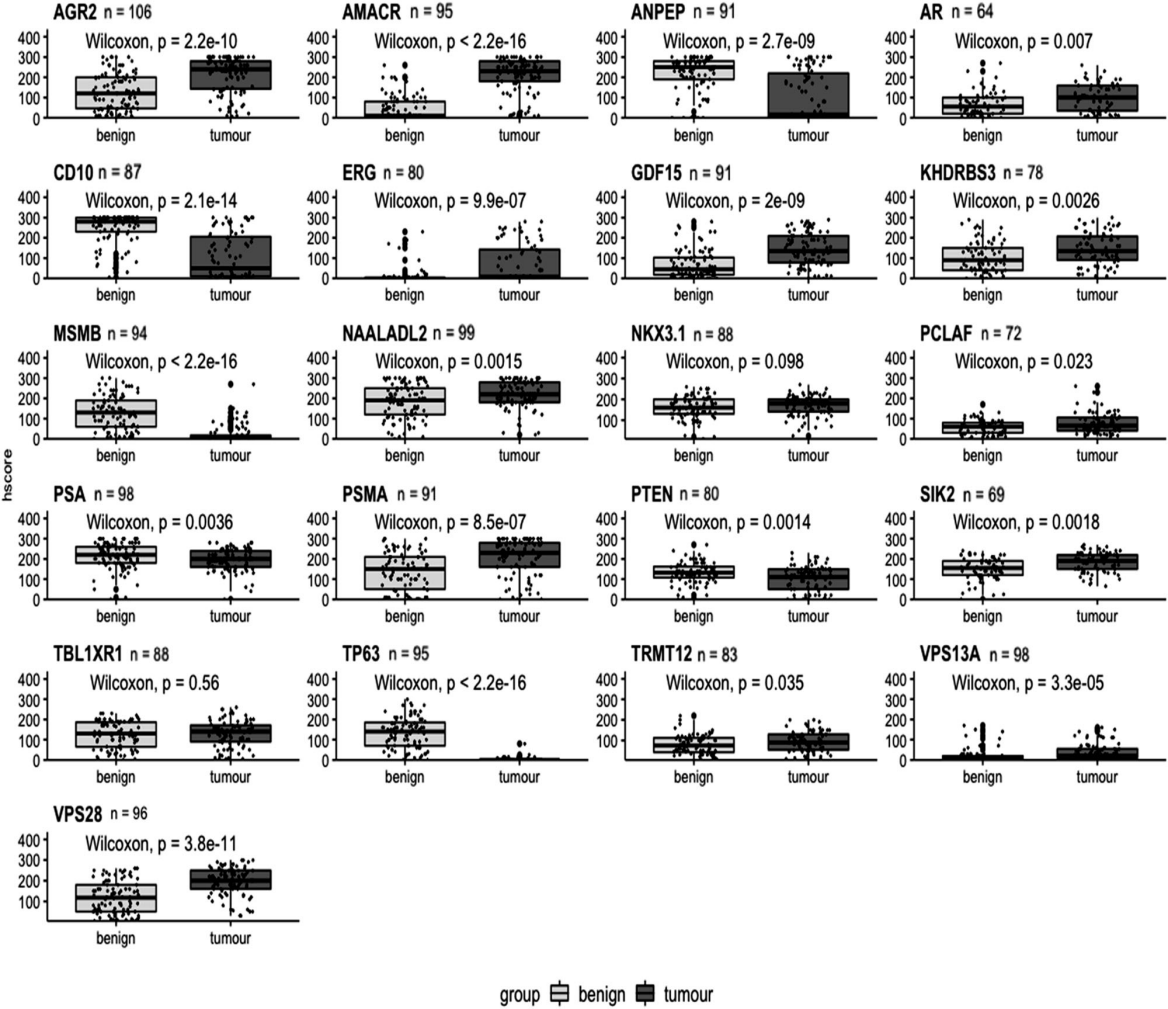
Expression of biomarkers differentiates benign and cancerous tissue. Boxplots comparing matched benign and cancer samples based on h-score for each biomarker. Benign tissue plotted as light grey; cancer tissue as dark grey. H-score plotted on *y*-axis. Benign and cancer plotted on *x*-axis. Biomarker name above each boxplot with *n* = number of patients. A Wilcoxon signed rank test was performed and *p* values shown on each graph.

Matched tissue from 249 patients underwent IHC staining for biomarkers previously associated with malignancy (Fig. 1). The h-scores for each marker, based on the intensity and number of positively stained cells, are shown in Fig. 2. Boxplots show the h-scores in cancer and adjacent benign tissue while the density plots show the distribution of the mean h-score between cancer and patient-matched benign tissue (Fig. 2 and Supplementary Fig. 1).

Using the Wilcoxon signed-rank test only two markers, NKX3.1 and TBL1XR1, were not statistically significant (Fig. 2). The remaining 19 biomarkers did show significantly different expression at the protein level between tumour and benign tissue; AGR2, AMACR, ANPEP, CD10, ERG, GDF15, MSMB, TP63, PSMA, VPS13A and VPS28 highly significantly (*p* < 0.001) and AR, KDHRBS3, NAALADL2, PSA, PTEN and SIK2 having a significance of *p* < 0.01. PCLAF and TRMT12 had a significance of *p* < 0.05. In the density plots, 13 biomarkers showed a significant difference between benign and malignant tissue: AGR2, AMACR, ANPEP, AR, CD10, ERG, GDF15, KHDRBS3, MSMB, NAALADL2, TP63, PSA, PSMA, SIK2, TRMT12 VPS13A and VPS28. Other biomarkers showed no significant difference between the mean h-score values; PCLAF, NKX3.1, PTEN and TBL1XR1 (Supplementary Fig. 1).

### Identification of biomarkers that can distinguish pathological grading

**Fig. 3:**
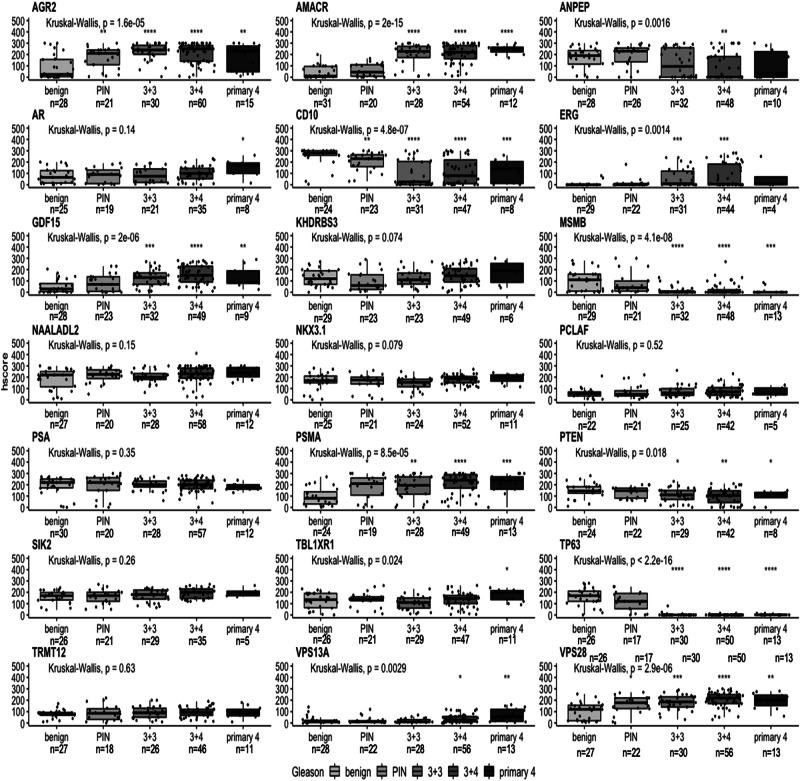
Distribution of biomarker h-scores in IHC compared to pathological Gleason grading in malignant tissue. Boxplots comparing the IHC h-score with Gleason grades in malignant tissue for each biomarker. The pathological comparison is based on patients’ biopsies classified as true benign (benign), prostatic intraepithelial neoplasia (PIN) and Gleason grading system: 3 + 3, 3 + 4 and primary 4 (4 + 3, 4 + 4 and 4 + 5). Pathological groups plotted on *x*-axis. H-score plotted on *y*-axis. Biomarker name above each boxplot graph. Number of patients (*n*) is displayed below each pathological group. Kruskal-Wallis rank sum test was performed with *p* value displayed above each boxplot. Each Gleason group was compared to true benign group (benign), a reference group, with *t*-test with *p < 0.05, **p < 0.01, ***p < 0.001 and ****p < 0.0001.

To assess the relationship between h-score and prostate pathology the highest Gleason score (i.e. the most aggressive) and/or the longest MCCL for each patient was compared. Analysis of h-scores of different pathological groups, identified 13 biomarkers in malignant tissue that were highly statistically significant (Kruskal-Wallis rank sum *p* ≤ 0.001) in comparison to true benign tissue: AGR2, AMACR, ANPEP, CD10, ERG, GDF15, MSMB, PSMA, PTEN, TBL1XR1, TP63, VPS13A and VPS28 (Fig. 3). Of these 13 biomarkers, at least one pathological group within the marker showed significant difference between Gleason grades compared to true benign group with AGR2, CD10, PSMA and VPS28 showing significance in all pathological groups (Fig. 3). The remaining identified markers were significantly different between ≤2 Gleason groups.

For the matched benign cores, AGR2, CD10 and ERG showed the highest degree of significance (Supplementary Fig. 2). Among those markers, a post hoc analysis showed that only AGR2 had a significant difference in all four pathological groups when compared to the true benign group. No significant correlation was shown for the rest of the biomarkers in adjacent benign cores.

### Identification of biomarkers that show clinically significant PCa on mpMRI

**Fig. 4:**
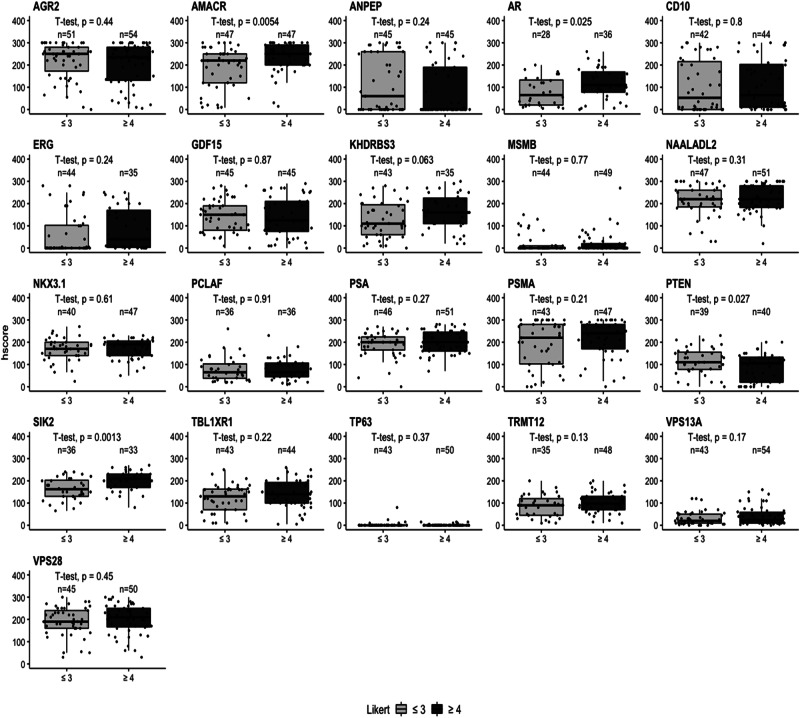
Biomarker h-scores and mpMRI imaging data to detect clinically significant PCa in malignant tissue. Boxplots comparing the IHC h-scores with MRI Likert grade in malignant tissue for each biomarker. The radiological comparison is split into two groups: Likert ≤ 3 (‘lower’ or ‘equivocal’ likelihood) and Likert ≥ 4 (‘higher’ likelihood). Likert groups are plotted on *x*-axis (light grey = Likert ≤ 3 and dark grey = Likert ≥ 4) while h-score is plotted on *y*-axis. Number of patients per group is plotted above each category. Biomarker name above each boxplot graph. Student’s *t*-test was performed with *p* value displayed above each boxplot.

As the visibility of cancer on mpMRI correlates with higher Gleason grade and the presence of clinically significant cancer, we investigated if the h-score correlated with mpMRI conspicuity. The Likert score and biomarker h-score were compared in matched samples based on two groups: less conspicuous, Likert ≤ 3 (‘lower’ and/or ‘equivocal’ likelihood of a clinically significant cancer diagnosis) and more conspicuous Likert ≥ 4 (‘higher’ likelihood of a clinically significant cancer diagnosis). In both malignant and adjacent benign cores, AMACR was identified as highly significant marker with *p* values of 0.0054 and 0.034, respectively (Fig. 4 and Supplementary Fig. 3). SIK2 (*p* = 0.0013), AR (*p* = 0.025) and PTEN (*p* = 0.027) also showed a significant difference in expression at the protein level detected by IHC in malignant tissue. The rest of the biomarkers showed no statistical difference between malignant and adjacent benign tissue (Fig. 4 and Supplementary Fig. 3).

The h-score data from patient-matched benign/tumour cores was also analysed by individual Likert group with AMACR and SIK2 showing significant differences of *p* values of 0.04 and 0.016, respectively in a malignant tissue; while in the benign tissue VPS13A (*p* = 0.028), ANPEP (*p* = 0.047) and SIK2 (*p* = 0.041) were significantly different among individual Likert groups (Supplementary Figs. 4 and 5). There were no significant changes between malignant and adjacent benign tissue for the remaining biomarkers (Supplementary Figs. 4 and 5).

Further to this, we also looked at the individual biomarker’s expression between clinically insignificant and clinically significant PCa based on the biopsy grading groups, GG1 and GG2 (clinically insignificant), and ≥GG3 (clinically significant), which was reported in other study [15]. This was performed on matched tumour biopsies only (Supplementary Fig. 6).

The results showed a significant correlation in NKX3.1 (*p* = 0.022 for GG1 vs GG2, and *p* = 0.035 for GG1 vs ≥GG3), TBL1XR1 (*p* = 0.0037 for GG1 vs ≥GG3) and VPS13A (*p* = 0.039 for GG1 vs GG2 and *p* = 0.007 for GG1 vs ≥GG3). Additionally, we looked at how the biopsy grading groups correlated in lower and higher Likert groups. For this reason, we grouped ≤Likert 3 and ≥Likert 4 and compared it with biopsy groups, GG1, GG2 and ≥GG3. This was based on the h-score in the matched tumour biopsies. Only SIK2 showed a significant correlation between Likert scores and GG2 group (*p* = 0.0029); however, no correlation for the remain groups. A marginal statistical significance was observed in KHDRBS3 and PTEN for Likert scores in GG2 group with *p* = 0.059 and *p* = 0.056, respectively. In abovementioned markers the expression difference was present in the group GG2 when comparing with lower and higher Likert score (Likert ≤3 and ≥4). Unfortunately, due to the small number of observations specifically in ≥GG3 group for most of the markers, the analysis was not successful. Therefore, no statistical significance was observed (Supplementary Fig. 7).

### Level of serum PSA in suspected PCa patients and the distribution of biomarker h-scores

For the analysis serum PSA was divided into three categories: <10 ng/mL, 10–15 ng/mL and >15 ng/mL and h-score data compared in both matched benign and tumour tissue for each patient. In malignant tissue, CD10 and PCLAF h-scores altered significantly with serum PSA (*p* = 0.0011 and 0.04 respectively, Supplementary Fig. 8). In the matched benign data, only tissue PSMA was significantly different (*p* = 0.00046) (Supplementary Fig. 9). The remaining biomarkers showed no significant difference between serum PSA level and biomarker expression in either malignant and matched benign tissue.

### Changes in biomarker expression in comparison to different age groups

As age is associated with an increased risk of PCa and poor prognosis we assessed how biomarker expression altered with age [16, 17]. Patients' age was stratified into three groups: <60, 60–70 and >70 and compared to biomarker h-score. Among the 21 markers, only GDF15 was significantly associated with age, using a Kruskal-Wallis rank sum test, in both malignant and matched benign tissue (*p* values 0.034 and 0.031, respectively, Supplementary Figs. 10 and 11).

### Changes in biomarker expression in patients with familial prostate cancer

We assessed if biomarker protein expression correlated with a reported family history of cancer [16, 17] but there was no statistical difference between groups of patients with or without family history for all biomarkers in the malignant tissue (Supplementary Fig. 12). However, GDF15 and SIK2 expression showed an increase in the adjacent benign tissue of men with a positive family history in comparison to patients with no family history, with *p* values of 0.0025 and 0.022, respectively (Supplementary Fig. 13).

## Discussion

The major advantage of this study lies in the comprehensive availability of the image mpMRI data with a representative core for the most aggressive cancer based on the template biopsy and also the clinicopathological variabilities (pathological assessment, level of serum PSA, age, ethnicity and familial PCa) of each patient. Consequently, the objective of this investigation was to identify biomarkers that demonstrate a diagnostic utility in both malignant and the surrounding adjacent benign tissues in relation to the imaging data. We performed IHC to look at the staining intensities of the markers in prostatic biopsies and linked it with the available clinical data. Our results confirmed the literature findings for almost all of the protein markers, where the staining was either over- or under-intensified in the benign and malignant tissues (Figs. 1, 2 and Supplementary Fig. 1). Two of the biomarkers with no changes between benign and malignant samples were NKX3.1 and TBL1XR1. This could be due to the antibodies used in the study or the biological variability and the heterogenous expression level of these proteins.

**Fig. 5:**
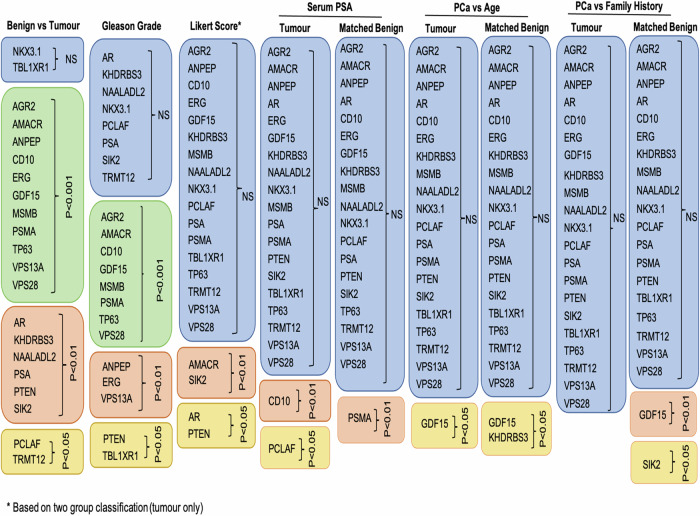
Summary table of 21 tissue markers tested in prostatic biopsies. Table shows summary of the results that tested 21 tissue biomarkers on prostatic malignant and matched, neighboburing benign tissues across different clinical data: benign versus tumour tissue, Gleason grade, Likert score, serum PSA, age and family history. Boxes in blue colour show no significant results, boxes in green show *p* < 0.0001, boxes in pink *p* < 0.01 and boxes in yellow *p* < 0.05. Likert score is based on the two-group classification (Fig. 4).

We compared results across various clinicopathological data (due to the limited number of data points, the analysis for ethnicity groups was omitted) in both types of tissue (Fig. 5). Examining the marker’s expression in adjacent benign samples is important as it can provide essential information about the tumour microenvironment, supporting diagnosis, prognosis and in better treatment options. Alterations in the morphology and microenvironment of neighbouring benign sample may suggest a presence of tumour-promoting agents that facilitate growth, invasion and metastasis of malignant cells [18, 19].

By examining the results of pathological evaluations and mpMRI Likert scores, we have identified two biomarkers, AMACR and PTEN, that share both diagnostic and prognostic value in PCa with AMACR being already used in clinical settings. Both biomarkers are associated with aggressive form of PCa and biochemical recurrence. Furthermore, their expression levels correlate with a higher Gleason grade and stage of the disease [20–22].

The majority of the biomarkers tested did not correlate with mpMRI Likert scores. However, CD10, KHDRBS3, PCLAF, PSMA, SIK2 and GDF15 were differentially expressed with PCa progression, emphasising their roles in promoting tumour growth, invasion and metastasis [22–28]. The upregulation of these biomarkers has been associated with poor prognosis and a more aggressive form of cancer suggesting a potentially important role in the disease [22–28].

Apart from the pathological Gleason assessment and mpMRI data, we investigated the biopsy GG system, comparing clinically insignificant (GG1 = Gleason 3 + 3 and GG2 = Gleason 3 + 4) and clinically significant (≥GG3 = ≥ Gleason score 4 + 3) PCa with Likert score. A notable finding emerged, revealing SIK2 as a biomarker showing statistical significance between Likert ≤ 3 and Likert ≥ 4 in biopsy grade group 2.

SIK2 is involved in regulating lipid metabolism and synthesis and is associated with cell survival and cell cycle regulation which is also enriched in high Gleason tumours [29]. The marker is upregulated and linked to a more aggressive disease phenotype.

While SIK2 has been implicated in metabolic pathways, its specific association with imaging in PCa remains less understood. Given its involvement in metabolic pathways and potential roles in cancer progression, SIK2 expression may indirectly influence tissue characteristics, aiding in risk stratification, treatment selection and prognosis assessment, thus positioning SIK2 as a promising prognostic marker and drug target.

Additionally, a marginal correlation was also observed in PTEN and KHDRBS3 markers in biopsy grade group 2 when comparing to Likert score, with their association to more aggressive tumours discussed earlier.

While limited data are available on the correlation between malignant tissue and its matched neighbouring benign tissue, some studies have shown that an elevated level of GDF15 in tumour tissue, compared to adjacent benign regions, has been found to increase the risk of PCa recurrence. This was also concomitant with higher Gleason grade [28, 30]. Interestingly, another study found elevated GDF15 in both pathologies, with slightly higher expression levels in adjacent benign regions [31]. These findings highlight the complexity of the tumour microenvironment and the need for further research to fully comprehend their role in cancer progression and modulating the tumour microenvironment.

Tissue markers in combination with mpMRI are a powerful tool in characterisation of clinically significant and potentially aggressive PCa. The incorporation of these biomarkers into diagnostic and prognostic tools can enhance the accuracy of PCa detection and prediction of disease outcomes. The biomarkers associated with poor prognosis and more aggressive forms of PCa can guide the development of targeted therapies and help management of the disease. However, better understanding the mechanisms of action is needed to maximise any therapeutic opportunities. The results presented here unveil tissue biomarkers that correlate with image data. However, further validation in imaging cohorts is required to ensure reproducibility. Given the changing face of PCa diagnosis and the routine incorporation of imaging within the diagnostic pathway, these results highlight the importance of using clinically relevant cohorts in biomarker validation studies at the earliest opportunity.

## Supplementary information

Supplementary material

## Data Availability

Correspondence and requests for materials and data generated in this study should be addressed to USF.
