# Supplementary material for: Combining tissue biomarkers with mpMRI to diagnose clinically significant prostate cancer. Analysis of 21 biomarkers in the PICTURE study
Source: Prostate Cancer Prostatic Dis. 2024 Nov 22;28(2):457–68. doi: 10.1038/s41391-024-00920-1 (PMC12106085; doi:10.1038/s41391-024-00920-1)
Supplement: Supplementary file 1 — Supplementary material [file 41391_2024_920_MOESM1_ESM.pptx]

## Slide 1
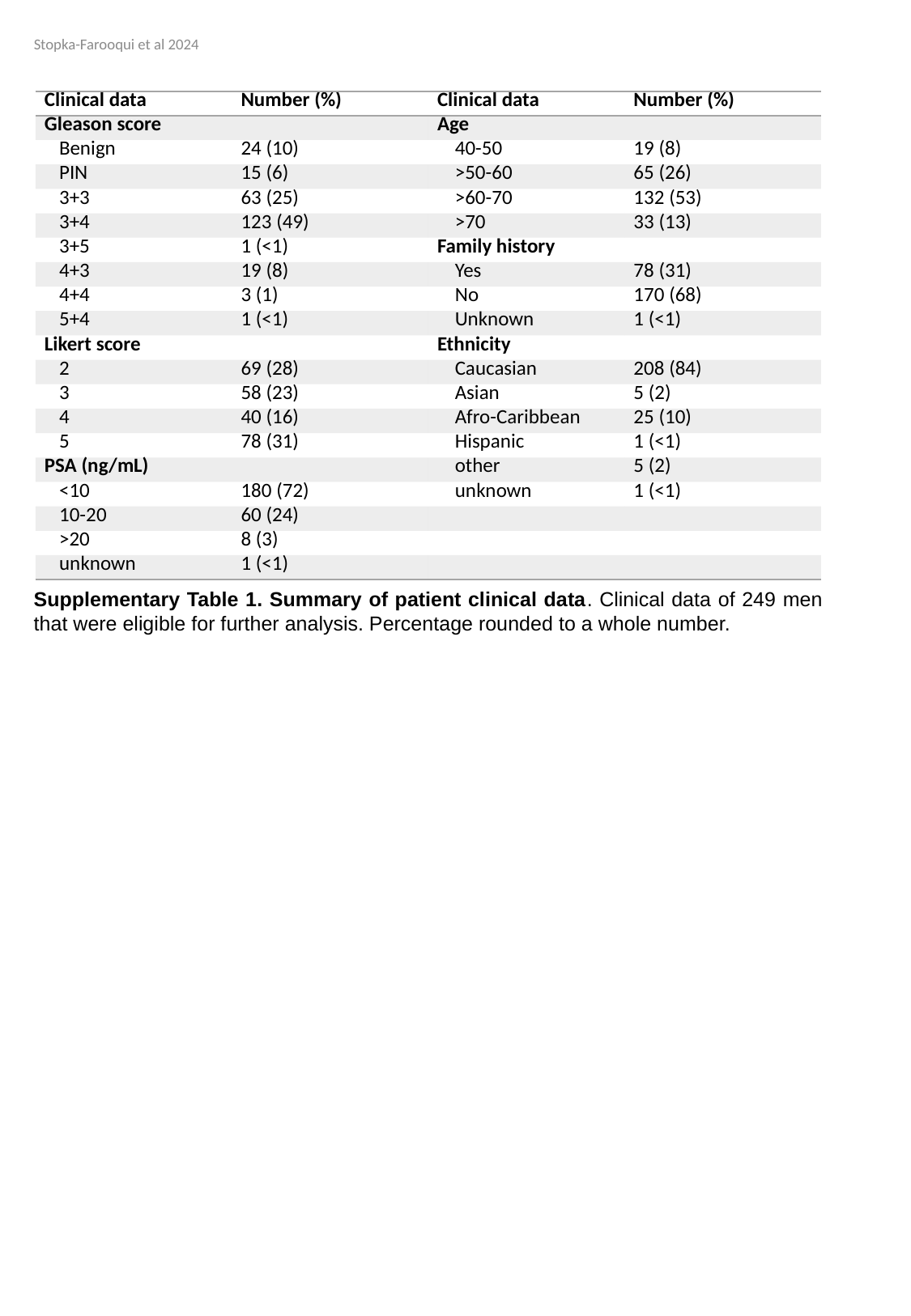

Stopka-Farooqui et al 2024
| Clinical data | Number (%) | Clinical data | Number (%) |
| --- | --- | --- | --- |
| Gleason score | | Age | |
| Benign | 24 (10) | 40-50 | 19 (8) |
| PIN | 15 (6) | >50-60 | 65 (26) |
| 3+3 | 63 (25) | >60-70 | 132 (53) |
| 3+4 | 123 (49) | >70 | 33 (13) |
| 3+5 | 1 (<1) | Family history | |
| 4+3 | 19 (8) | Yes | 78 (31) |
| 4+4 | 3 (1) | No | 170 (68) |
| 5+4 | 1 (<1) | Unknown | 1 (<1) |
| Likert score | | Ethnicity | |
| 2 | 69 (28) | Caucasian | 208 (84) |
| 3 | 58 (23) | Asian | 5 (2) |
| 4 | 40 (16) | Afro-Caribbean | 25 (10) |
| 5 | 78 (31) | Hispanic | 1 (<1) |
| PSA (ng/mL) | | other | 5 (2) |
| <10 | 180 (72) | unknown | 1 (<1) |
| 10-20 | 60 (24) | | |
| >20 | 8 (3) | | |
| unknown | 1 (<1) | | |
Supplementary Table 1. Summary of patient clinical data. Clinical data of 249 men that were eligible for further analysis. Percentage rounded to a whole number.

## Slide 2
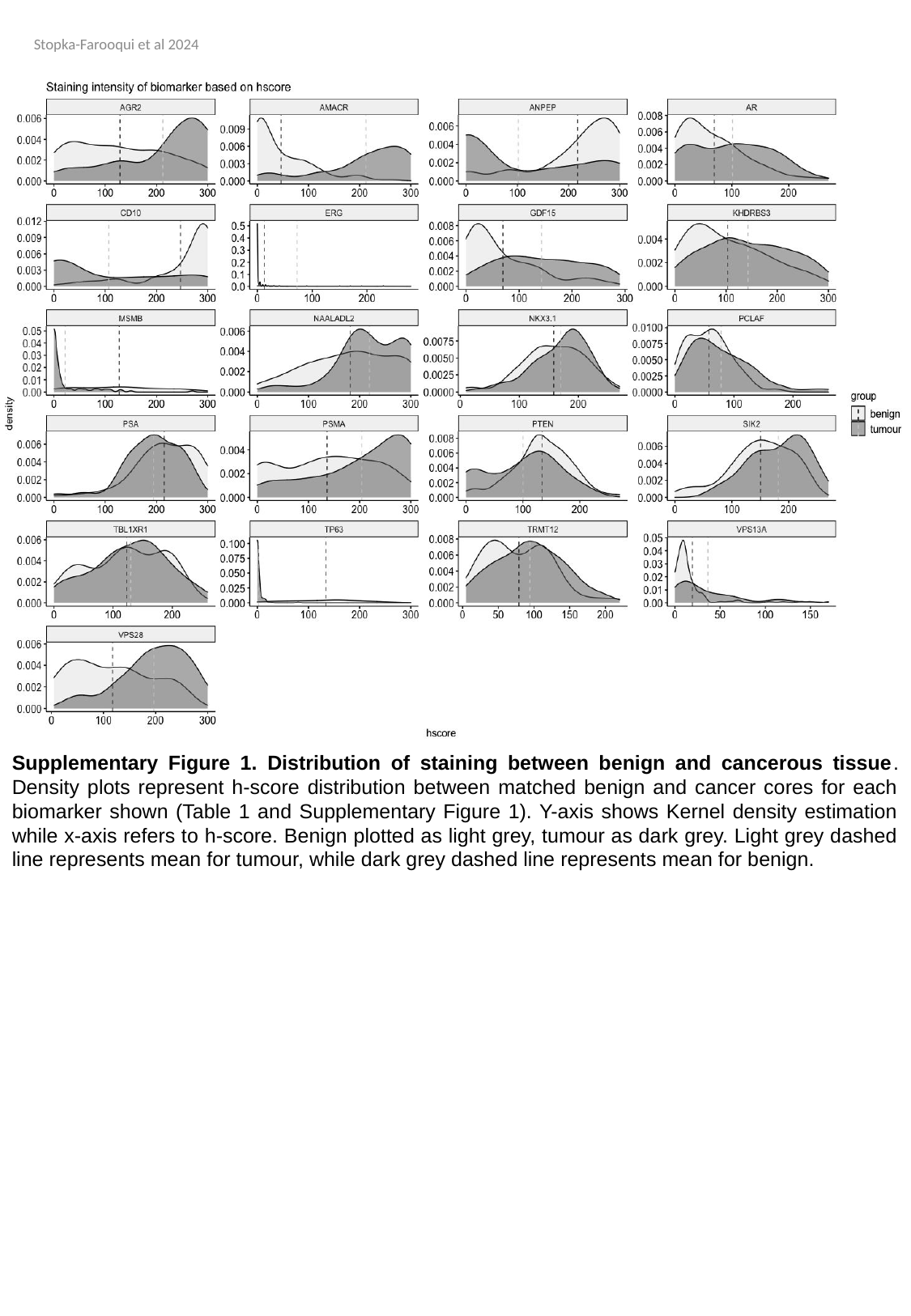

Stopka-Farooqui et al 2024
Supplementary Figure 1. Distribution of staining between benign and cancerous tissue. Density plots represent h-score distribution between matched benign and cancer cores for each biomarker shown (Table 1 and Supplementary Figure 1). Y-axis shows Kernel density estimation while x-axis refers to h-score. Benign plotted as light grey, tumour as dark grey. Light grey dashed line represents mean for tumour, while dark grey dashed line represents mean for benign.

## Slide 3
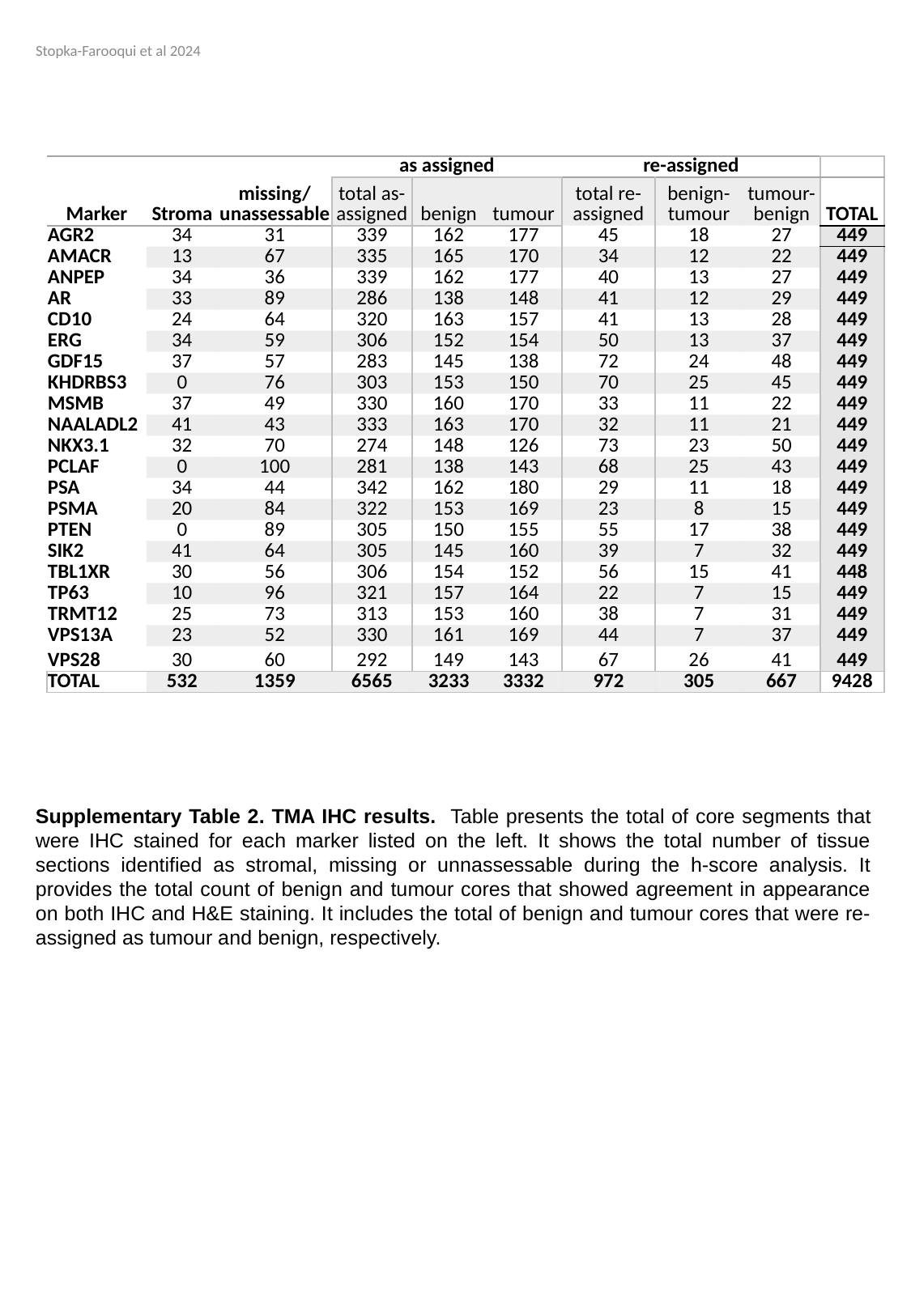

Stopka-Farooqui et al 2024
| Marker | Stroma | missing/ unassessable | as assigned | | | re-assigned | | | |
| --- | --- | --- | --- | --- | --- | --- | --- | --- | --- |
| | | | total as-assigned | benign | tumour | total re-assigned | benign-tumour | tumour-benign | TOTAL |
| AGR2 | 34 | 31 | 339 | 162 | 177 | 45 | 18 | 27 | 449 |
| AMACR | 13 | 67 | 335 | 165 | 170 | 34 | 12 | 22 | 449 |
| ANPEP | 34 | 36 | 339 | 162 | 177 | 40 | 13 | 27 | 449 |
| AR | 33 | 89 | 286 | 138 | 148 | 41 | 12 | 29 | 449 |
| CD10 | 24 | 64 | 320 | 163 | 157 | 41 | 13 | 28 | 449 |
| ERG | 34 | 59 | 306 | 152 | 154 | 50 | 13 | 37 | 449 |
| GDF15 | 37 | 57 | 283 | 145 | 138 | 72 | 24 | 48 | 449 |
| KHDRBS3 | 0 | 76 | 303 | 153 | 150 | 70 | 25 | 45 | 449 |
| MSMB | 37 | 49 | 330 | 160 | 170 | 33 | 11 | 22 | 449 |
| NAALADL2 | 41 | 43 | 333 | 163 | 170 | 32 | 11 | 21 | 449 |
| NKX3.1 | 32 | 70 | 274 | 148 | 126 | 73 | 23 | 50 | 449 |
| PCLAF | 0 | 100 | 281 | 138 | 143 | 68 | 25 | 43 | 449 |
| PSA | 34 | 44 | 342 | 162 | 180 | 29 | 11 | 18 | 449 |
| PSMA | 20 | 84 | 322 | 153 | 169 | 23 | 8 | 15 | 449 |
| PTEN | 0 | 89 | 305 | 150 | 155 | 55 | 17 | 38 | 449 |
| SIK2 | 41 | 64 | 305 | 145 | 160 | 39 | 7 | 32 | 449 |
| TBL1XR | 30 | 56 | 306 | 154 | 152 | 56 | 15 | 41 | 448 |
| TP63 | 10 | 96 | 321 | 157 | 164 | 22 | 7 | 15 | 449 |
| TRMT12 | 25 | 73 | 313 | 153 | 160 | 38 | 7 | 31 | 449 |
| VPS13A | 23 | 52 | 330 | 161 | 169 | 44 | 7 | 37 | 449 |
| VPS28 | 30 | 60 | 292 | 149 | 143 | 67 | 26 | 41 | 449 |
| TOTAL | 532 | 1359 | 6565 | 3233 | 3332 | 972 | 305 | 667 | 9428 |
Supplementary Table 2. TMA IHC results. Table presents the total of core segments that were IHC stained for each marker listed on the left. It shows the total number of tissue sections identified as stromal, missing or unnassessable during the h-score analysis. It provides the total count of benign and tumour cores that showed agreement in appearance on both IHC and H&E staining. It includes the total of benign and tumour cores that were re-assigned as tumour and benign, respectively.

## Slide 4
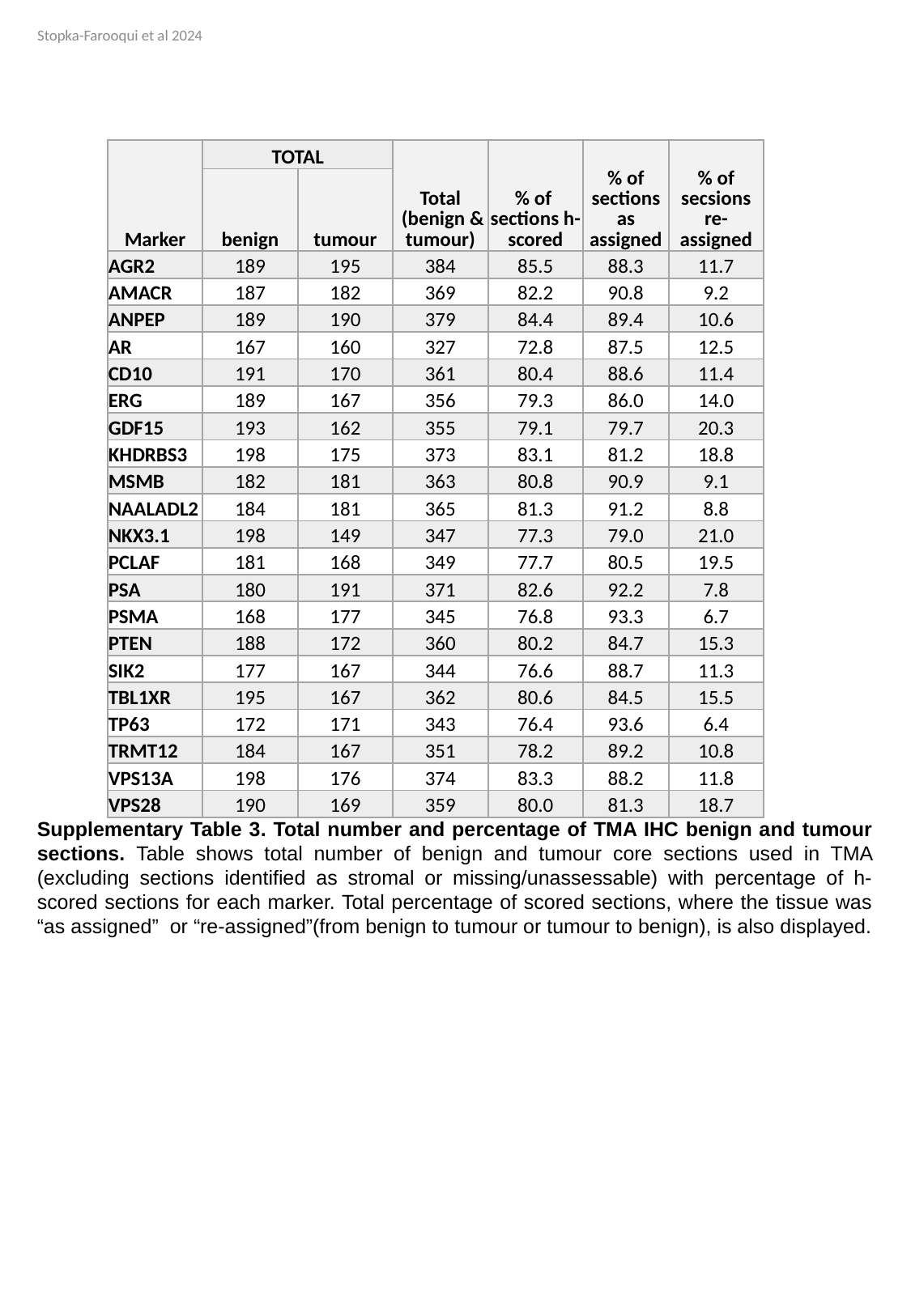

Stopka-Farooqui et al 2024
| Marker | TOTAL | | Total (benign & tumour) | % of sections h-scored | % of sections as assigned | % of secsions re-assigned |
| --- | --- | --- | --- | --- | --- | --- |
| Marker | benign | tumour | | | | |
| AGR2 | 189 | 195 | 384 | 85.5 | 88.3 | 11.7 |
| AMACR | 187 | 182 | 369 | 82.2 | 90.8 | 9.2 |
| ANPEP | 189 | 190 | 379 | 84.4 | 89.4 | 10.6 |
| AR | 167 | 160 | 327 | 72.8 | 87.5 | 12.5 |
| CD10 | 191 | 170 | 361 | 80.4 | 88.6 | 11.4 |
| ERG | 189 | 167 | 356 | 79.3 | 86.0 | 14.0 |
| GDF15 | 193 | 162 | 355 | 79.1 | 79.7 | 20.3 |
| KHDRBS3 | 198 | 175 | 373 | 83.1 | 81.2 | 18.8 |
| MSMB | 182 | 181 | 363 | 80.8 | 90.9 | 9.1 |
| NAALADL2 | 184 | 181 | 365 | 81.3 | 91.2 | 8.8 |
| NKX3.1 | 198 | 149 | 347 | 77.3 | 79.0 | 21.0 |
| PCLAF | 181 | 168 | 349 | 77.7 | 80.5 | 19.5 |
| PSA | 180 | 191 | 371 | 82.6 | 92.2 | 7.8 |
| PSMA | 168 | 177 | 345 | 76.8 | 93.3 | 6.7 |
| PTEN | 188 | 172 | 360 | 80.2 | 84.7 | 15.3 |
| SIK2 | 177 | 167 | 344 | 76.6 | 88.7 | 11.3 |
| TBL1XR | 195 | 167 | 362 | 80.6 | 84.5 | 15.5 |
| TP63 | 172 | 171 | 343 | 76.4 | 93.6 | 6.4 |
| TRMT12 | 184 | 167 | 351 | 78.2 | 89.2 | 10.8 |
| VPS13A | 198 | 176 | 374 | 83.3 | 88.2 | 11.8 |
| VPS28 | 190 | 169 | 359 | 80.0 | 81.3 | 18.7 |
Supplementary Table 3. Total number and percentage of TMA IHC benign and tumour sections. Table shows total number of benign and tumour core sections used in TMA (excluding sections identified as stromal or missing/unassessable) with percentage of h-scored sections for each marker. Total percentage of scored sections, where the tissue was “as assigned” or “re-assigned”(from benign to tumour or tumour to benign), is also displayed.

## Slide 5
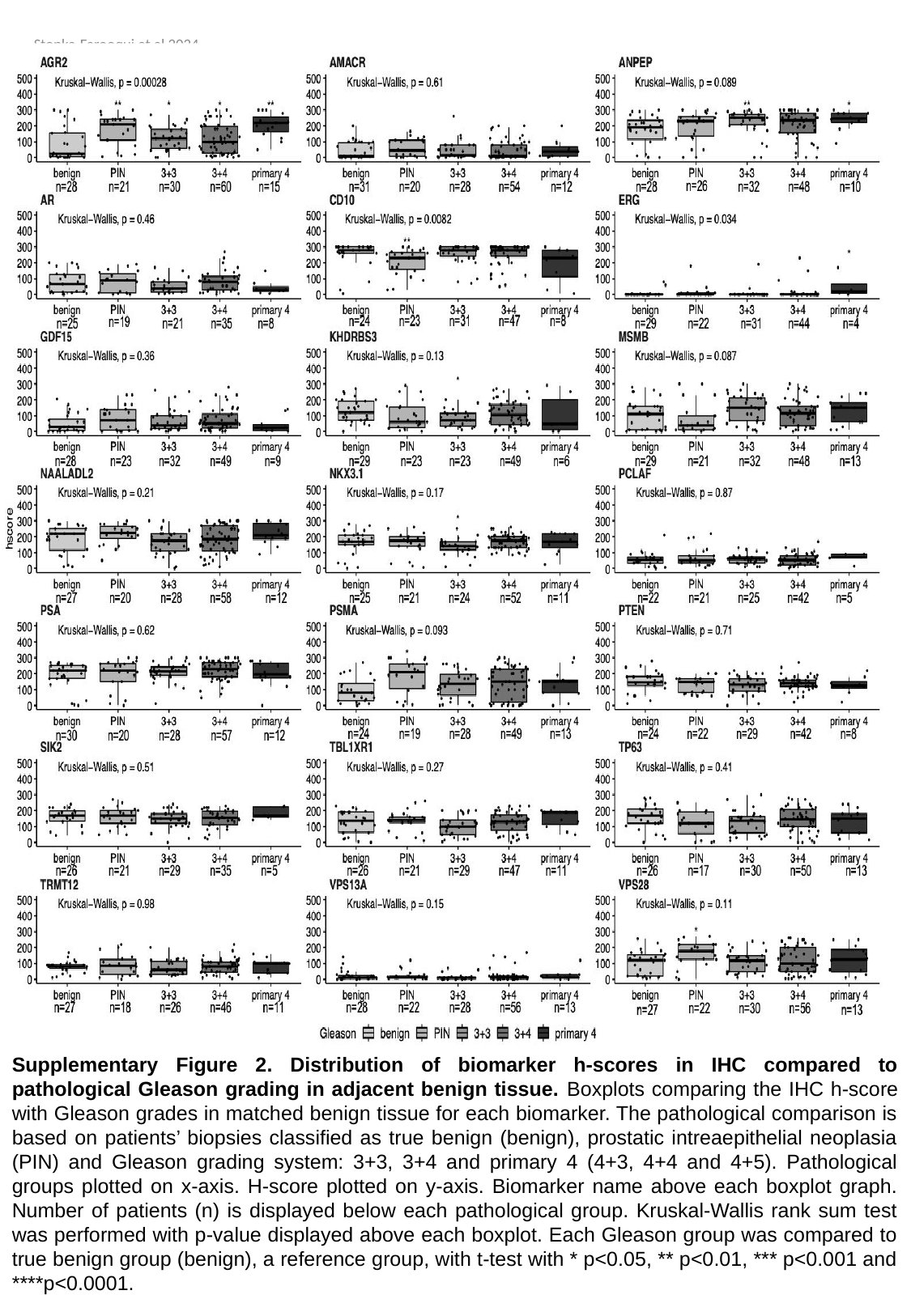

Stopka-Farooqui et al 2024
Supplementary Figure 2. Distribution of biomarker h-scores in IHC compared to pathological Gleason grading in adjacent benign tissue. Boxplots comparing the IHC h-score with Gleason grades in matched benign tissue for each biomarker. The pathological comparison is based on patients’ biopsies classified as true benign (benign), prostatic intreaepithelial neoplasia (PIN) and Gleason grading system: 3+3, 3+4 and primary 4 (4+3, 4+4 and 4+5). Pathological groups plotted on x-axis. H-score plotted on y-axis. Biomarker name above each boxplot graph. Number of patients (n) is displayed below each pathological group. Kruskal-Wallis rank sum test was performed with p-value displayed above each boxplot. Each Gleason group was compared to true benign group (benign), a reference group, with t-test with * p<0.05, ** p<0.01, *** p<0.001 and ****p<0.0001.

## Slide 6
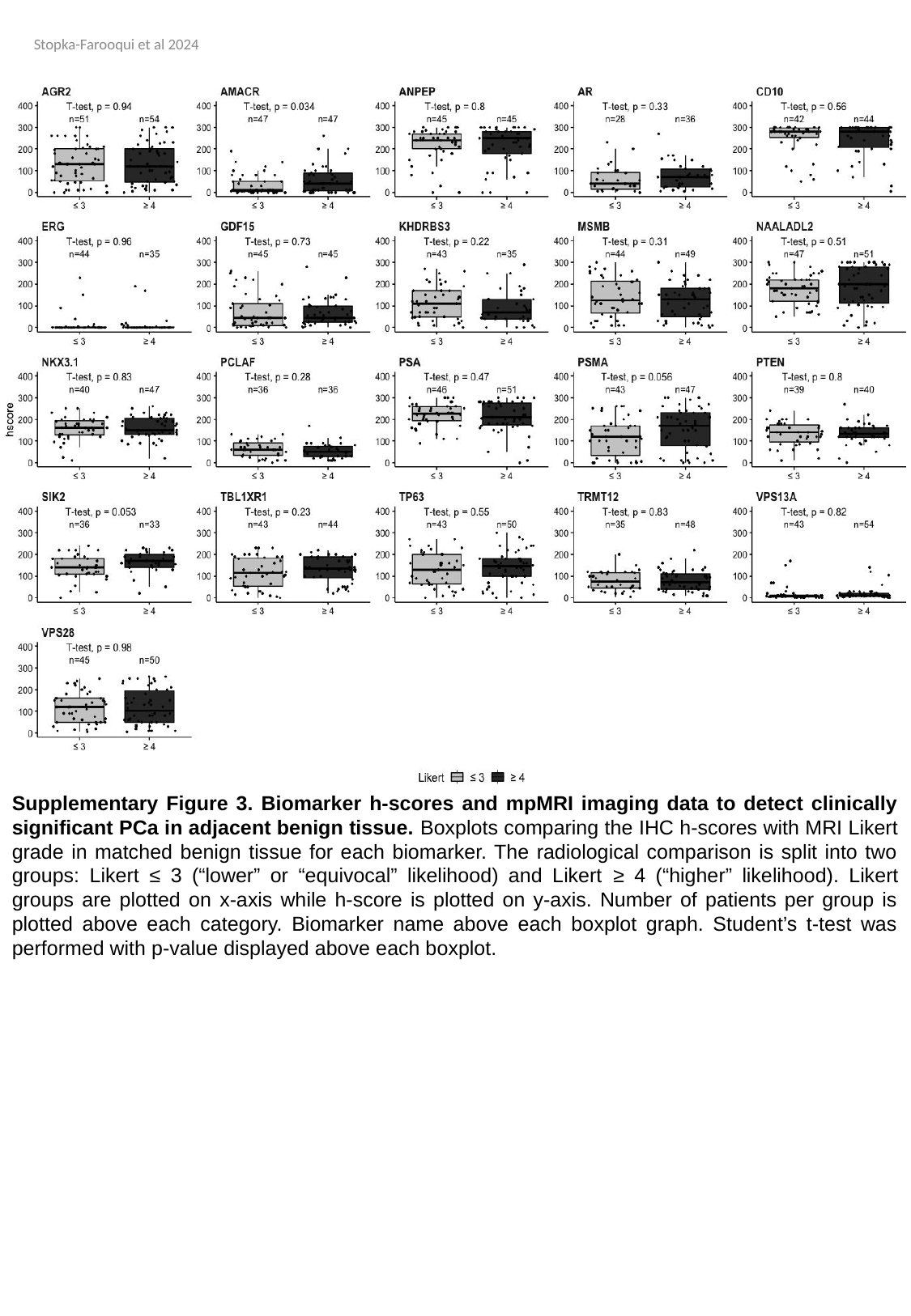

Stopka-Farooqui et al 2024
Supplementary Figure 3. Biomarker h-scores and mpMRI imaging data to detect clinically significant PCa in adjacent benign tissue. Boxplots comparing the IHC h-scores with MRI Likert grade in matched benign tissue for each biomarker. The radiological comparison is split into two groups: Likert ≤ 3 (“lower” or “equivocal” likelihood) and Likert ≥ 4 (“higher” likelihood). Likert groups are plotted on x-axis while h-score is plotted on y-axis. Number of patients per group is plotted above each category. Biomarker name above each boxplot graph. Student’s t-test was performed with p-value displayed above each boxplot.

## Slide 7
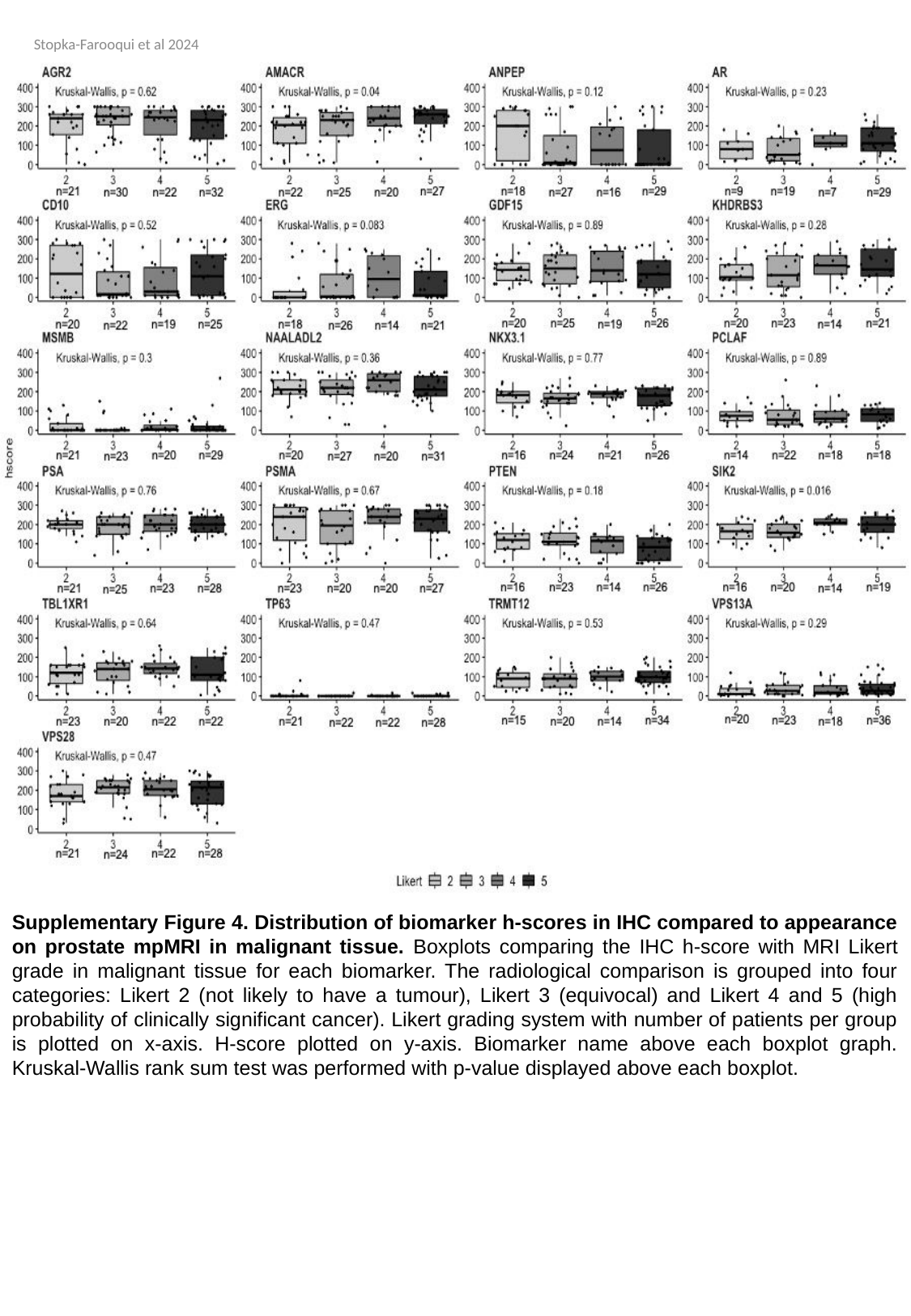

Stopka-Farooqui et al 2024
Supplementary Figure 4. Distribution of biomarker h-scores in IHC compared to appearance on prostate mpMRI in malignant tissue. Boxplots comparing the IHC h-score with MRI Likert grade in malignant tissue for each biomarker. The radiological comparison is grouped into four categories: Likert 2 (not likely to have a tumour), Likert 3 (equivocal) and Likert 4 and 5 (high probability of clinically significant cancer). Likert grading system with number of patients per group is plotted on x-axis. H-score plotted on y-axis. Biomarker name above each boxplot graph. Kruskal-Wallis rank sum test was performed with p-value displayed above each boxplot.

## Slide 8
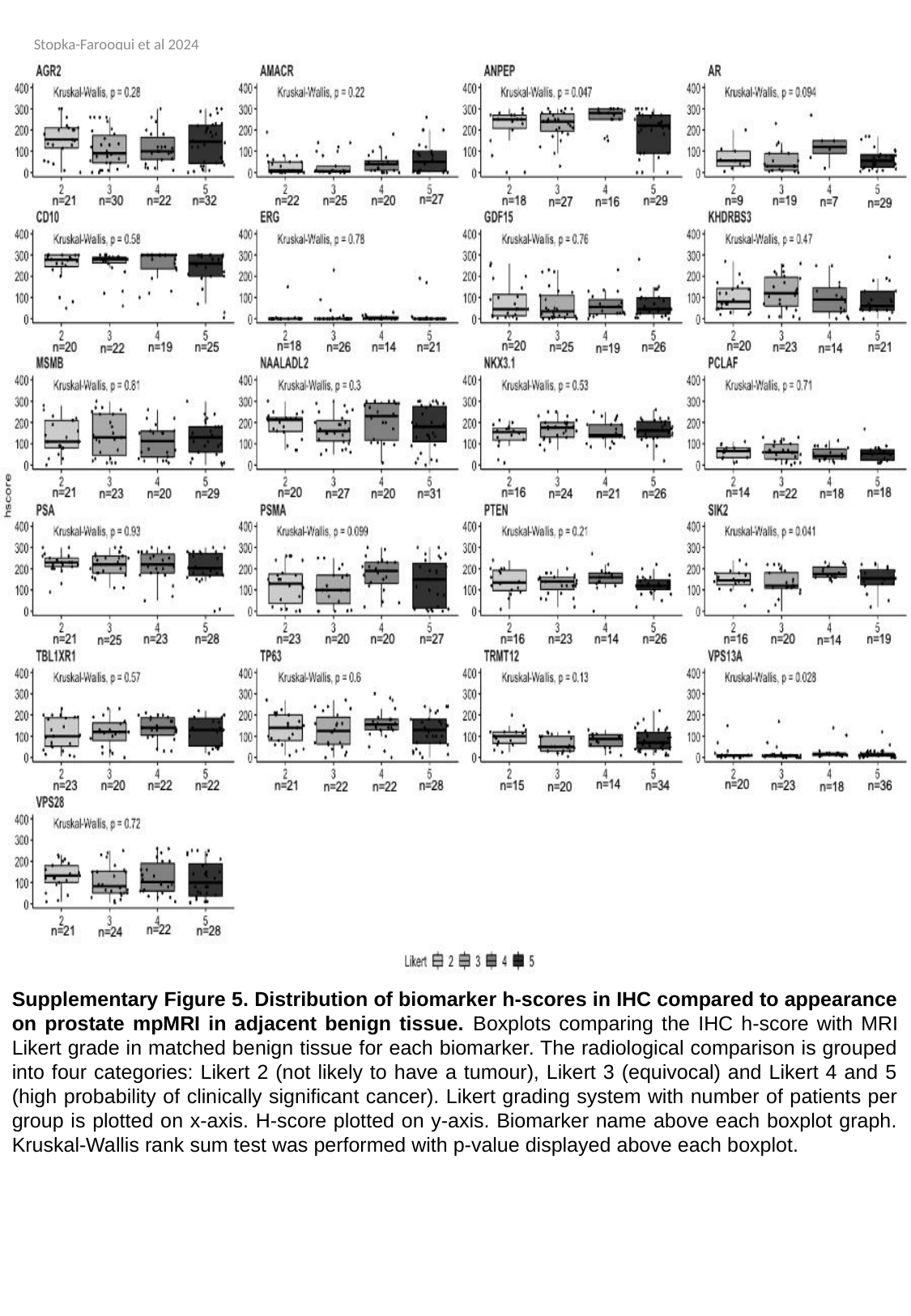

Stopka-Farooqui et al 2024
Supplementary Figure 5. Distribution of biomarker h-scores in IHC compared to appearance on prostate mpMRI in adjacent benign tissue. Boxplots comparing the IHC h-score with MRI Likert grade in matched benign tissue for each biomarker. The radiological comparison is grouped into four categories: Likert 2 (not likely to have a tumour), Likert 3 (equivocal) and Likert 4 and 5 (high probability of clinically significant cancer). Likert grading system with number of patients per group is plotted on x-axis. H-score plotted on y-axis. Biomarker name above each boxplot graph. Kruskal-Wallis rank sum test was performed with p-value displayed above each boxplot.

## Slide 9
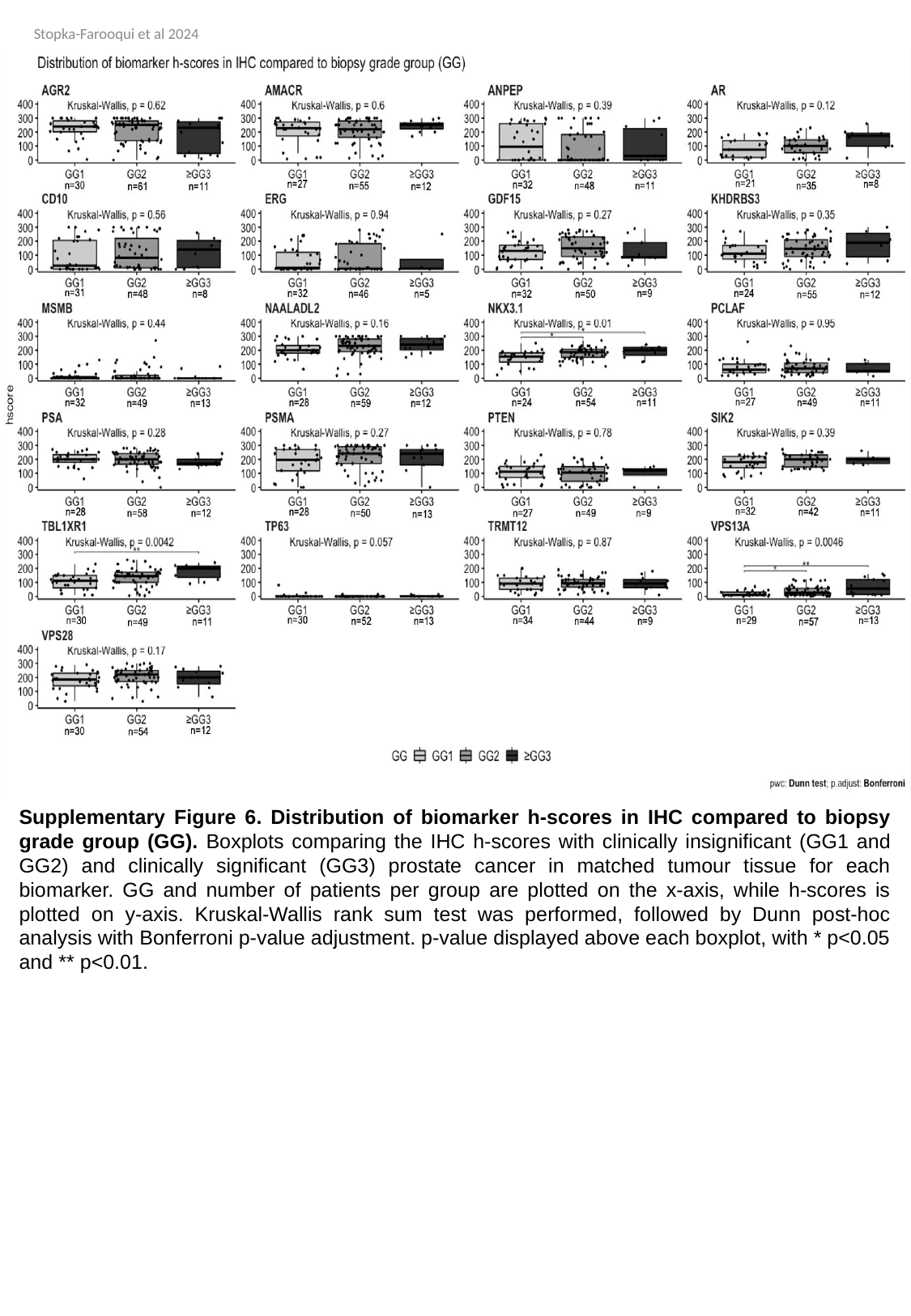

Stopka-Farooqui et al 2024

## Slide 10
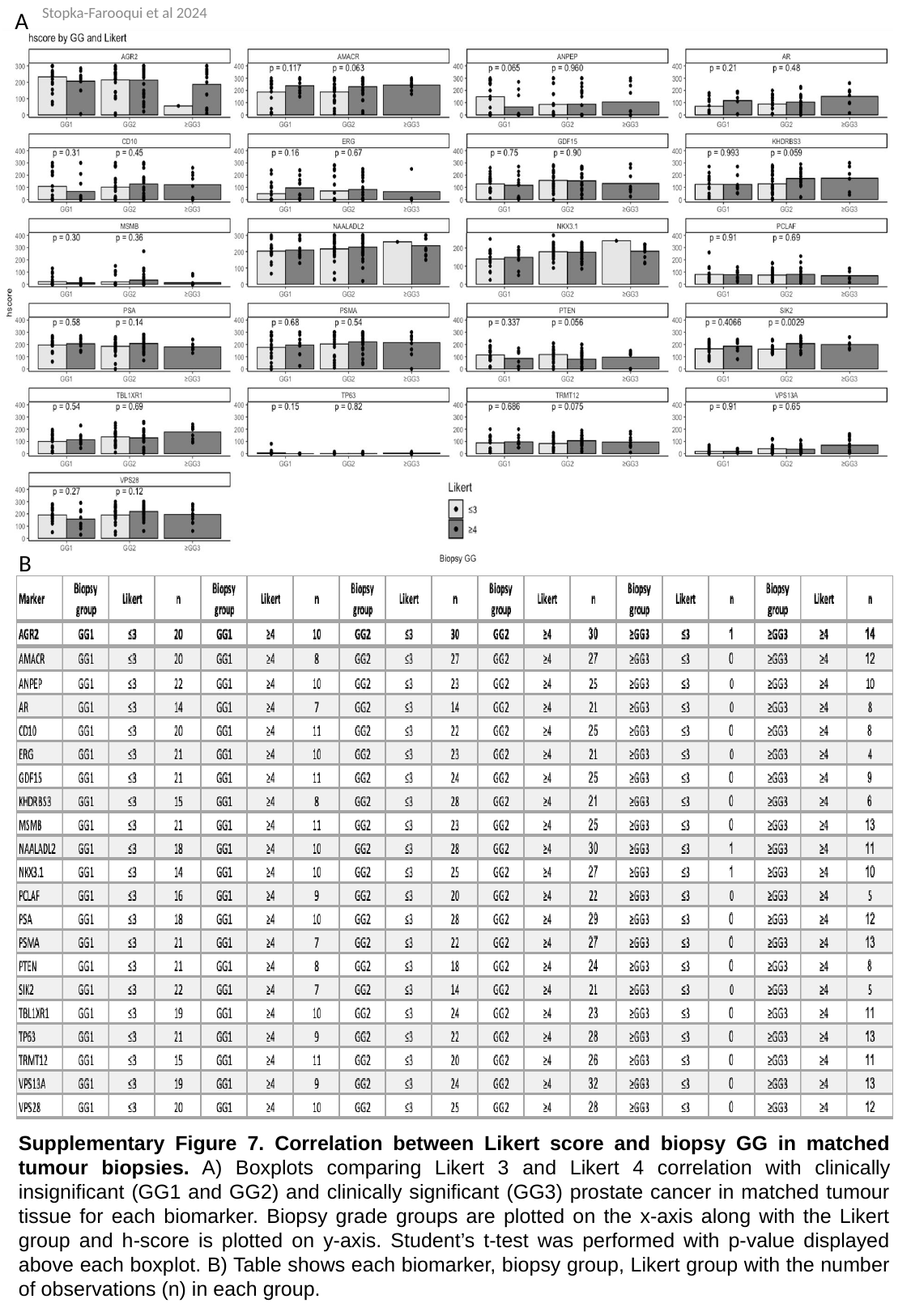

A
Stopka-Farooqui et al 2024
B

## Slide 11
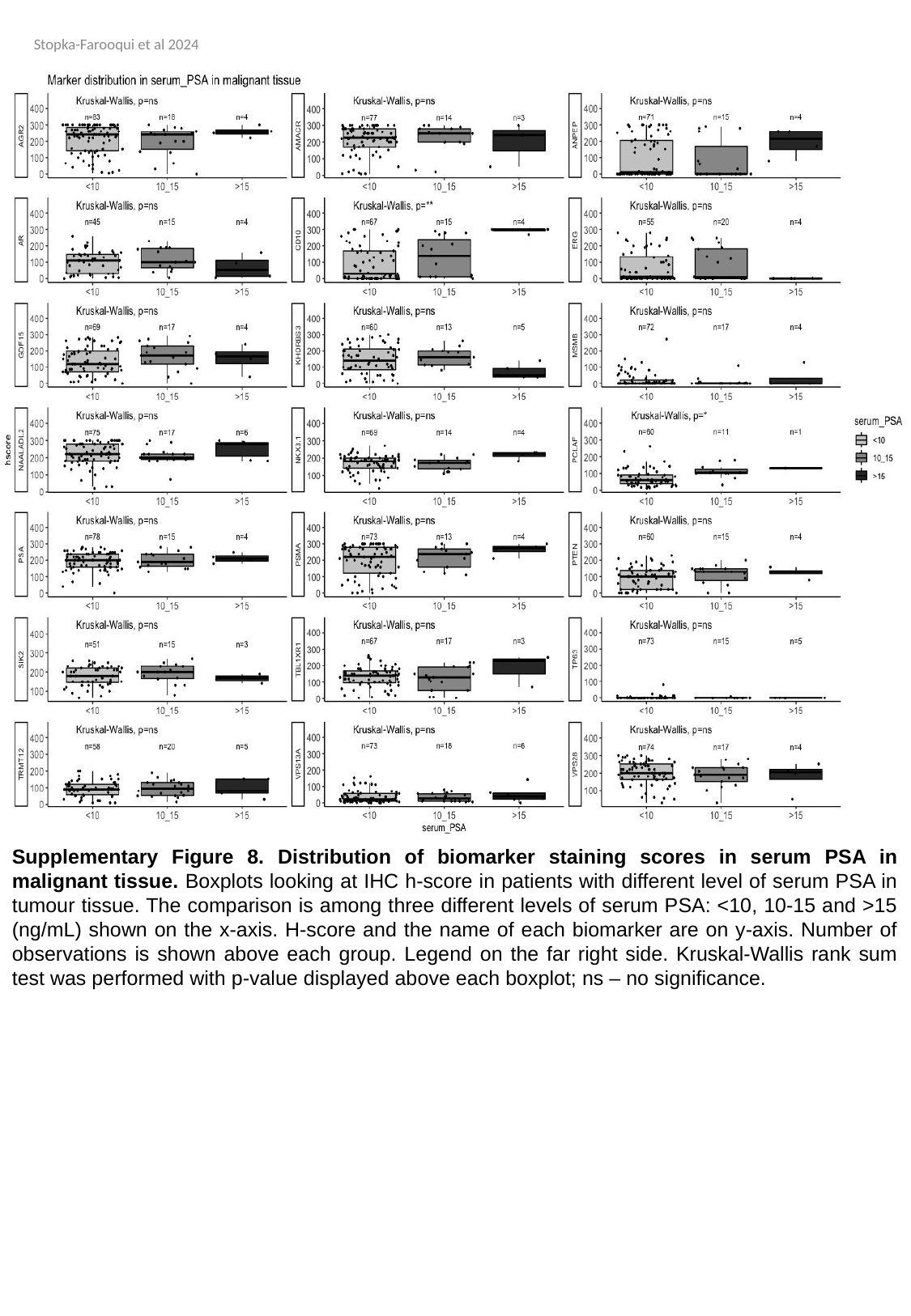

Stopka-Farooqui et al 2024
Supplementary Figure 8. Distribution of biomarker staining scores in serum PSA in malignant tissue. Boxplots looking at IHC h-score in patients with different level of serum PSA in tumour tissue. The comparison is among three different levels of serum PSA: <10, 10-15 and >15 (ng/mL) shown on the x-axis. H-score and the name of each biomarker are on y-axis. Number of observations is shown above each group. Legend on the far right side. Kruskal-Wallis rank sum test was performed with p-value displayed above each boxplot; ns – no significance.

## Slide 12
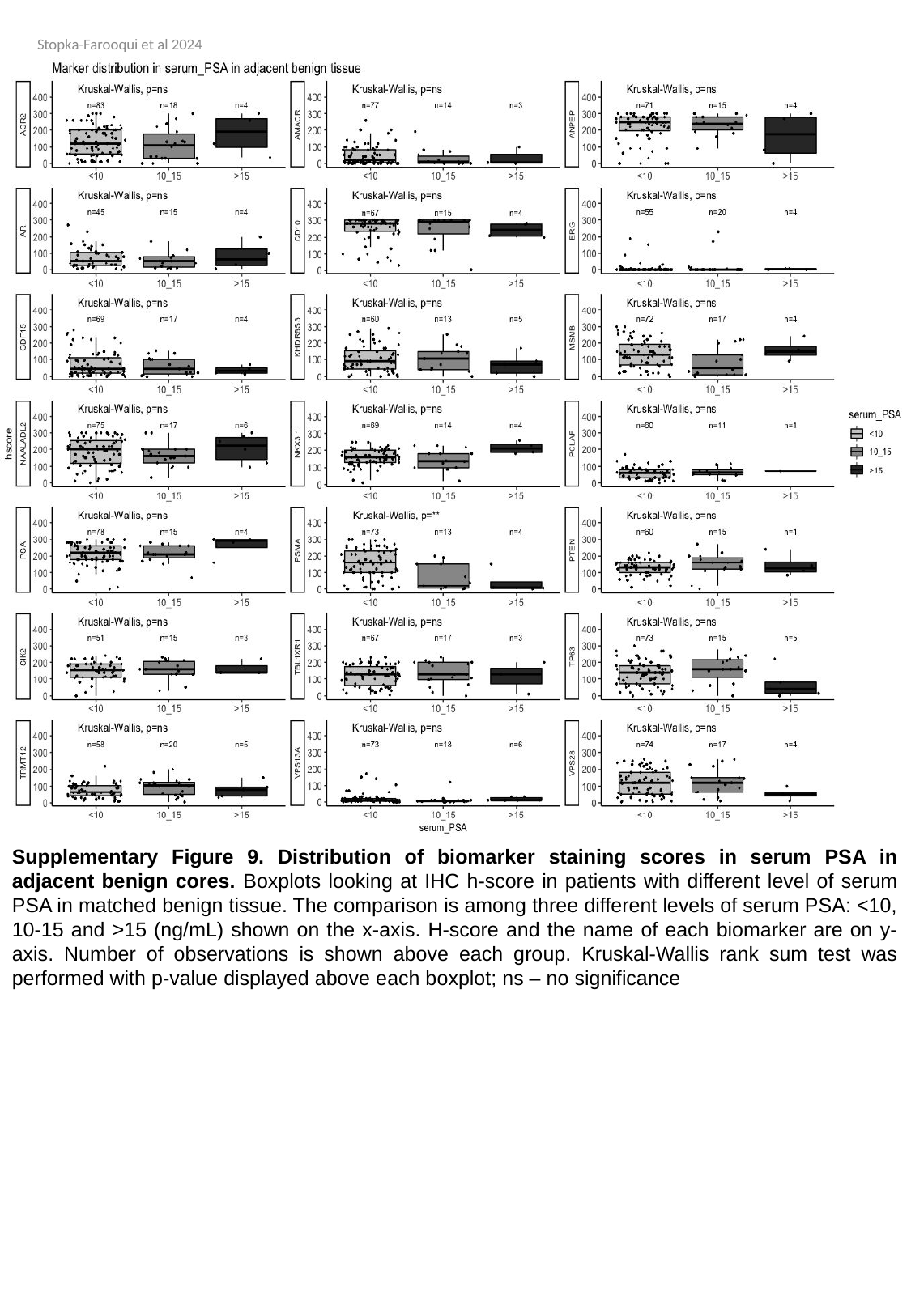

Stopka-Farooqui et al 2024
Supplementary Figure 9. Distribution of biomarker staining scores in serum PSA in adjacent benign cores. Boxplots looking at IHC h-score in patients with different level of serum PSA in matched benign tissue. The comparison is among three different levels of serum PSA: <10, 10-15 and >15 (ng/mL) shown on the x-axis. H-score and the name of each biomarker are on y-axis. Number of observations is shown above each group. Kruskal-Wallis rank sum test was performed with p-value displayed above each boxplot; ns – no significance

## Slide 13
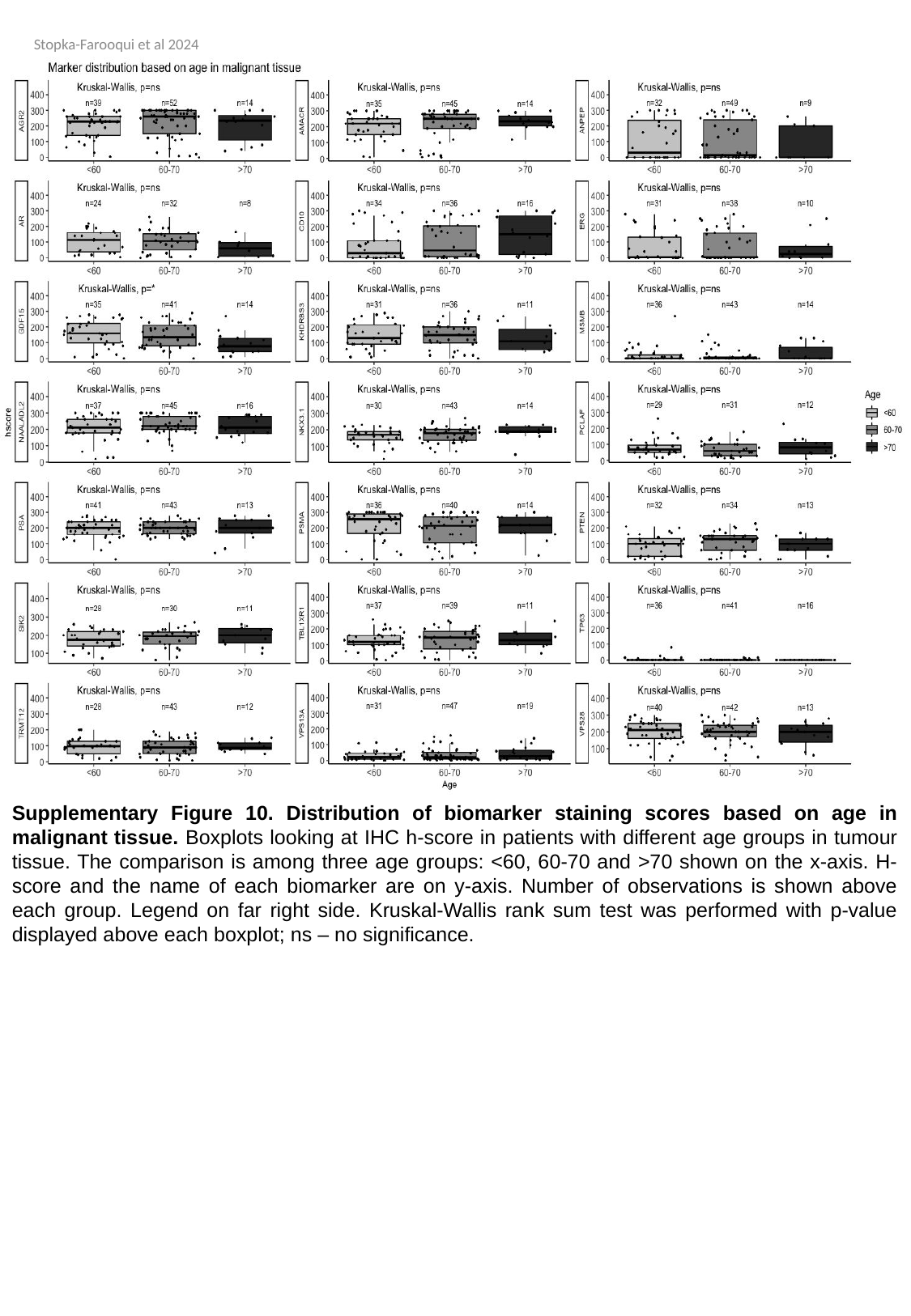

Stopka-Farooqui et al 2024
Supplementary Figure 10. Distribution of biomarker staining scores based on age in malignant tissue. Boxplots looking at IHC h-score in patients with different age groups in tumour tissue. The comparison is among three age groups: <60, 60-70 and >70 shown on the x-axis. H-score and the name of each biomarker are on y-axis. Number of observations is shown above each group. Legend on far right side. Kruskal-Wallis rank sum test was performed with p-value displayed above each boxplot; ns – no significance.

## Slide 14
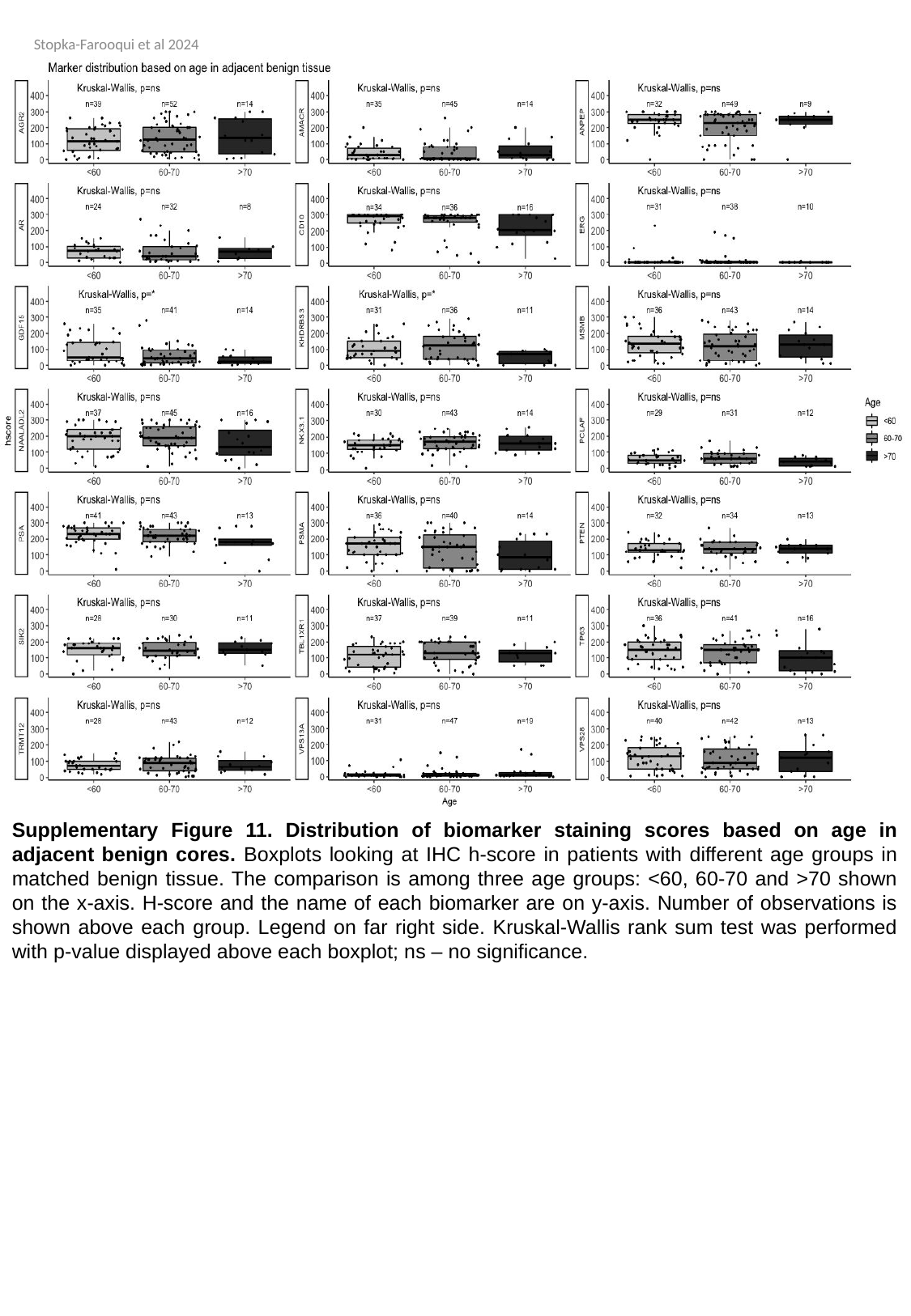

Stopka-Farooqui et al 2024
Supplementary Figure 11. Distribution of biomarker staining scores based on age in adjacent benign cores. Boxplots looking at IHC h-score in patients with different age groups in matched benign tissue. The comparison is among three age groups: <60, 60-70 and >70 shown on the x-axis. H-score and the name of each biomarker are on y-axis. Number of observations is shown above each group. Legend on far right side. Kruskal-Wallis rank sum test was performed with p-value displayed above each boxplot; ns – no significance.

## Slide 15
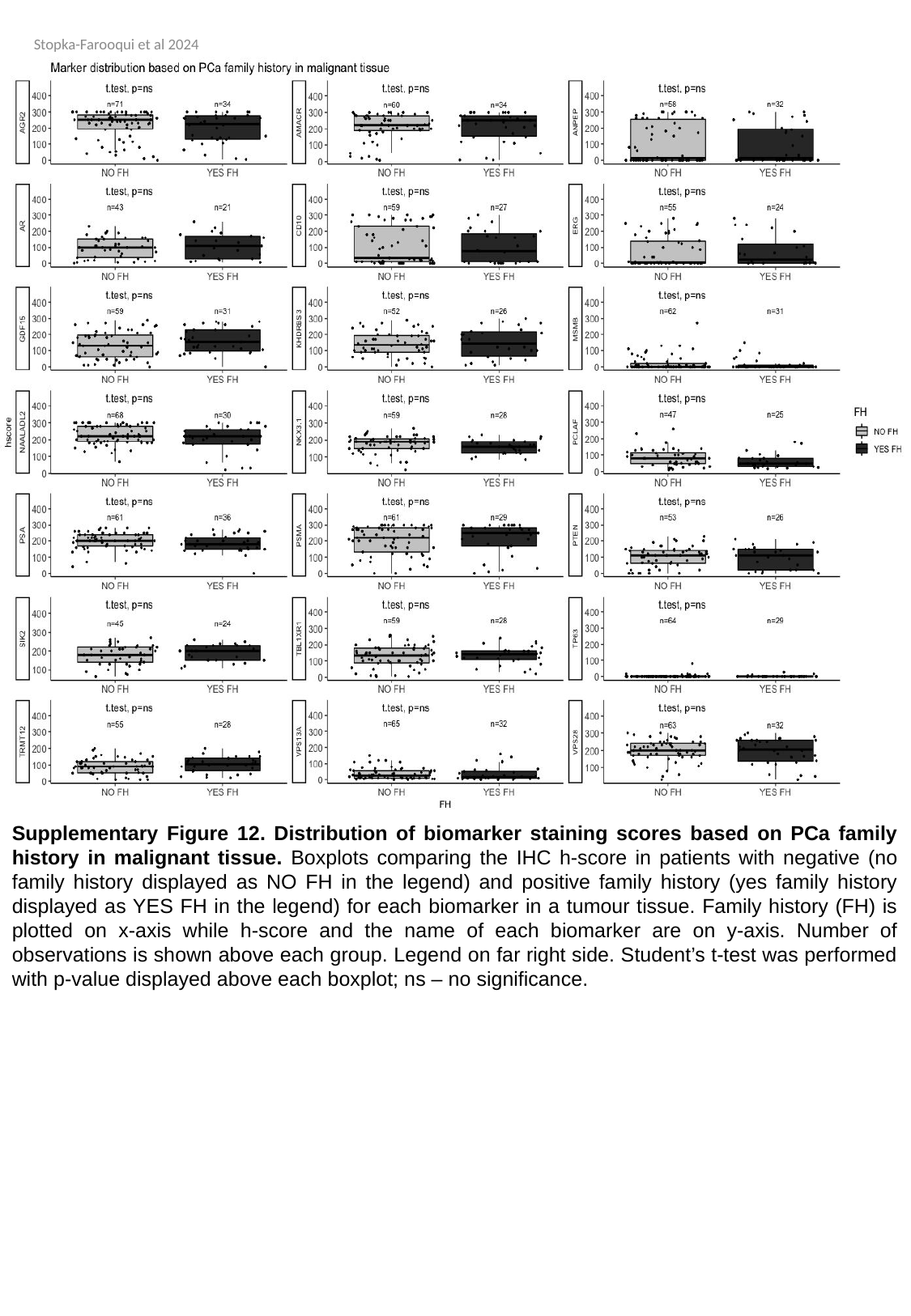

Stopka-Farooqui et al 2024
Supplementary Figure 12. Distribution of biomarker staining scores based on PCa family history in malignant tissue. Boxplots comparing the IHC h-score in patients with negative (no family history displayed as NO FH in the legend) and positive family history (yes family history displayed as YES FH in the legend) for each biomarker in a tumour tissue. Family history (FH) is plotted on x-axis while h-score and the name of each biomarker are on y-axis. Number of observations is shown above each group. Legend on far right side. Student’s t-test was performed with p-value displayed above each boxplot; ns – no significance.

## Slide 16
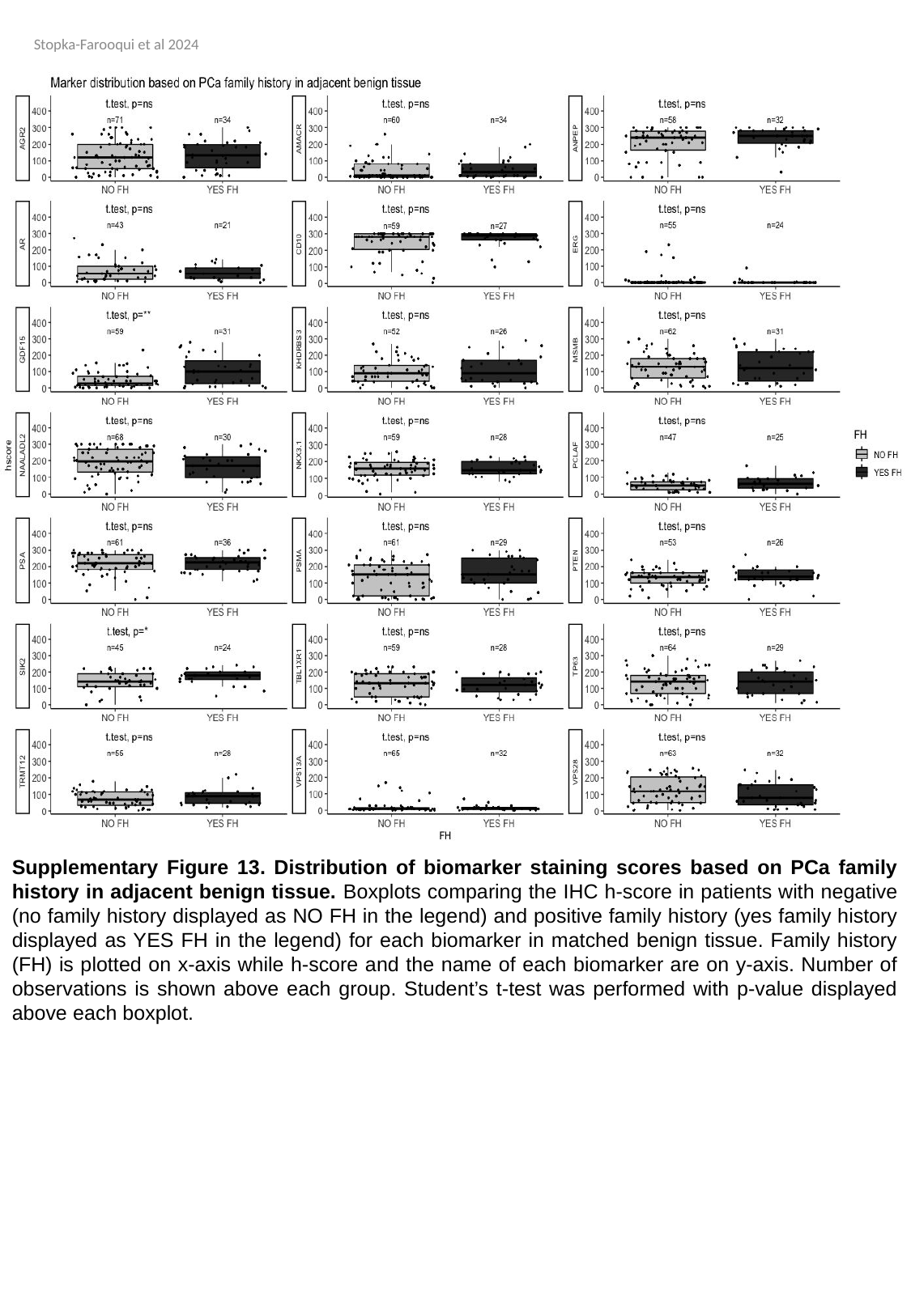

Stopka-Farooqui et al 2024
Supplementary Figure 13. Distribution of biomarker staining scores based on PCa family history in adjacent benign tissue. Boxplots comparing the IHC h-score in patients with negative (no family history displayed as NO FH in the legend) and positive family history (yes family history displayed as YES FH in the legend) for each biomarker in matched benign tissue. Family history (FH) is plotted on x-axis while h-score and the name of each biomarker are on y-axis. Number of observations is shown above each group. Student’s t-test was performed with p-value displayed above each boxplot.
